# Extended‐amygdala intrinsic functional connectivity networks: A population study

**DOI:** 10.1002/hbm.25314

**Published:** 2020-12-12

**Authors:** Samuel C. Berry, Richard G. Wise, Andrew D. Lawrence, Thomas M. Lancaster

**Affiliations:** ^1^ Cardiff University Brain Research Imaging Centre (CUBRIC), School of Psychology Cardiff University Cardiff UK; ^2^ Institute for Advanced Biomedical Technologies, Department of Neuroscience, Imaging and Clinical Sciences "G. D'Annunzio University" of Chieti‐Pescara Chieti Italy; ^3^ School of Psychology University of Bath Bath UK

**Keywords:** alcohol use, bed nucleus of the stria terminalis (BST/BNST), central nucleus of the amygdala (CeA), dispositional negativity, extended amygdala (ExtA), intrinsic functional connectivity (iFC), task‐free functional magnetic resonance imaging (tf‐fMRI)

## Abstract

Pre‐clinical and human neuroimaging research implicates the extended‐amygdala (ExtA) (including the bed nucleus of the stria terminalis [BST] and central nucleus of the amygdala [CeA]) in networks mediating negative emotional states associated with stress and substance‐use behaviours. The extent to which individual ExtA structures form a functionally integrated unit is controversial. We utilised a large sample (*n* > 1,000 healthy young adult humans) to compare the intrinsic functional connectivity networks (ICNs) of the BST and CeA using task‐free functional magnetic resonance imaging (fMRI) data from the Human Connectome Project. We assessed whether inter‐individual differences within these ICNs were related to two principal components representing negative disposition and alcohol use. Building on recent primate evidence, we tested whether within BST‐CeA intrinsic functional connectivity (iFC) was heritable and further examined co‐heritability with our principal components. We demonstrate the BST and CeA to have discrete, but largely overlapping ICNs similar to previous findings. We found no evidence that within BST—CeA iFC was heritable; however, post hoc analyses found significant BST iFC heritability with the broader superficial and centromedial amygdala regions. There were no significant correlations or co‐heritability associations with our principal components either across the ICNs or for specific BST‐Amygdala iFC. Possible differences in phenotype associations across task‐free, task‐based, and clinical fMRI are discussed, along with suggestions for more causal investigative paradigms that make use of the now well‐established ExtA ICNs.

## Graphical abstract

We examine the intrinsic connectivity networks of two key nodes within the extended amygdala in a large human sample. We find no associations within this network with principal components related to negative disposition or alcohol use traits. We report heritable intrinsic functional connections between the bed nucleus of the stria terminalis (BST) and the centromedial and superficial amygdala regions; however, do not repeat a recent non‐human primate finding of a heritable connection between the BST and central nucleus of the amygdala.

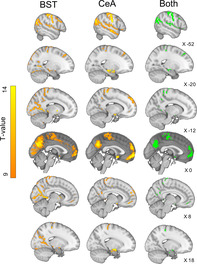

## 1 INTRODUCTION

The extended‐amygdala (ExtA) is a basal forebrain macrosystem that describes a set of small, complex and heterogenous subcortical nuclei between the amygdala and ventral striatum (Alheid et al., 1998; Alheid & Heimer, 1988; Alheid, 2009; Cassell, Freedman, & Shi, 1999; Fudge et al., 2017; Johnston, 1923). Its principal structures include the bed nucleus of the stria terminalis (BST) and the central nucleus of the amygdala (CeA), as well as portions of the shell of the nucleus accumbens and the sublenticular extended amygdala (SLEA) (an extension of amygdala neurons that connect the CeA and BST) (Alheid, 2009; Cassell et al., 1999; Fox, Oler, Tromp, Fudge, & Kalin, 2015; Fox & Shackman, 2019; Lebow & Chen, 2016; Martin, Powers, Dellovade, & Price, 1991; Stamatakis et al., 2014). This macrostructure, or neuronal continuum, has emerged as key area of interest in the investigation of anxiety, fear, and substance use (Ahrens et al., 2018; Avery, Clauss, & Blackford, 2016; Fox & Shackman, 2019; Gilpin, Herman, & Roberto, 2015; Goode, Ressler, Acca, Miles, & Maren, 2019; Goode & Maren, 2017; Lebow & Chen, 2016; Roberto, Kirson, & Khom, 2020; Stamatakis et al., 2014; Volkow, Koob, & McLellan, 2016).

Part of the interest in the ExtA stems from its anatomic location. With structural connections to areas including sensory, mnemonic, affective, and regulatory processing regions, the ExtA is strategically placed to coordinate activities in multiple “limbic lobe” areas for the development of behavioural responses through its output channels (Avery et al., 2016; Fox et al., 2015; Fox & Shackman, 2019; Heimer & Van Hoesen, 2006). As such, and in particular because of its direct outputs to the hypothalamic pituitary adrenal axis, it has been implicated in multiple behaviours linked to the processing of threat, stressors, and negative emotional states (Fox & Shackman, 2019; Giardino et al., 2018; Lebow & Chen, 2016).

That the ExtA is a key component within a stress‐related network further implicates it as an area of interest for substance‐use behaviours (Avery et al., 2016; Erikson, Wei, & Walker, 2018; Stamatakis et al., 2014; Volkow et al., 2016). Specifically, the ExtA is thought to be important in the dysphoric state associated with drug withdrawal and stress‐induced relapse and has been associated with cellular changes following alcohol use (Avery et al., 2016; Ch'ng, Fu, Brown, McDougall, & Lawrence, 2018; Erikson et al., 2018; Roberto et al., 2020; Stamatakis et al., 2014; Volkow et al., 2016). Association of the ExtA with both alcohol and anxiety is especially interesting given the high comorbidity between the two, with anxiety often precipitating alcohol use and being a hallmark of withdrawal (Gilpin et al., 2015). Experimental evidence for involvement in fear, anxiety, stress, and substance‐use derives from a multitude of lesion, optogenetic, and neural tracing studies in animals and, more recently, human neuroimaging studies (for reviews, see Ahrens et al., 2018; Avery et al., 2016; Ch'ng et al., 2018; Fox & Shackman, 2019; Goode, Acca, & Maren, 2020; Lebow & Chen, 2016).

Advances in neuroimaging techniques and the recent availability of high‐quality ExtA anatomical masks (Theiss, Ridgewell, McHugo, Heckers, & Blackford, 2017; Tillman et al., 2018; Torrisi et al., 2015; Tyszka & Pauli, 2016), have enabled several studies to use intrinsic functional connectivity (iFC) mapping of task‐free functional magnetic resonance imaging (tf‐fMRI) data to examine how ExtA activity is correlated with activity in other regions under resting conditions (Table 1) (Avery et al., 2014; Gorka, Torrisi, Shackman, Grillon, & Ernst, 2018; Hofmann & Straube, 2019; Motzkin et al., 2015; Oler et al., 2012; Tillman et al., 2018; Torrisi et al., 2015, 2019; Weis et al., 2019). This analysis approach allows researchers to identify “intrinsic connectivity networks” (ICNs) which can serve as an estimate of the brain's functional architecture at rest (Kelly & Castellanos, 2014; Seeley et al., 2007). The ICNs are highly organised, reproducible, and are similar to extrinsic (task‐driven) co‐activation patterns (Battistella et al., 2020; Suárez, Markello, Betzel, & Misic, 2020; Thomas Yeo et al., 2011). IFC is correlated with structural connectivity at around ~*R* ^2^ = .5 (Honey et al., 2009; Suárez et al., 2020). The remaining variance can be explained by co‐activation of regions with indirect connections that, for example, are two or more synapses removed from each other or between homotopic areas within each hemisphere that are not directly connected (Suárez et al., 2020).

**TABLE 1:** Previous human ExtA task‐free functional connectivity studies

Citation	Sample	*n*	Coverage	Native EPI resolution	Smoothing	Scanner (s)	CeA seed	BST seed	Technique
Oler et al. (2012)	Combination of three independent samples of adolescents and children (aged between 7.8 and 18 years old)	105	Whole brain	3 × 3 × 3 mm	6 mm	3 T Siemens Allegra, 3 T Siemens Magnetom Trio Tim, 3 T discovery MR750	Manually prescribed in right amygdala on a standard 152‐brain MRI template using ROI drawing tool in AFNI and Mai brain atlas	N/A	Voxel‐wise seed‐based correlation analysis
Avery et al. (2014)	Adults from ages 17 to 57 (*M* = 30.6, *SD*+−11.3) years old	99	Whole brain	3 × 3 × 4 mm	3 mm	3 T Phillips Achieva	N/A	Manually prescribed on 7 T anatomical GRASE image from a 42 year old Caucasian male.	Voxel‐wise and targeted region seed‐based correlation analysis
Motzkin et al. (2015)	Four adult vmPFC lesion patients and 19 healthy adults.	23	Whole brain	3.5 × 3.5 × 3 mm	4 mm	3 T discovery MR750	N/A	Manually prescribed on MNI template brain using Mai brain atlas	Cerebral blood flow case/control seed‐based correlation analysis
Torrisi et al. (2015)	Healthy adult volunteers (*M* = 27.3, *SD* = 6, years old)	27	Partial	1.3 × 1.3 × 1.3 mm	2.6 mm	7 T Siemens Magnetom	N/A	Manually prescribed by three raters on subjects structural images	Voxel‐wise seed‐based correlation analysis
Gorka et al. (2018)	Same as Torrisi et al.'s (2015) sample	27	Partial	1.3 × 1.3 × 1.3 mm	2.6 mm	7 T Siemens Magnetom	Mask from Tyszka and Pauli (2016) amygdala sub‐regions atlas. 20% thresholded	As prescribed in Torrisi et al. (2015)	Voxel‐wise seed‐based correlation analysis
Tillman et al. (2018)	Healthy adults from the NKI dataset (*M* = 25.3, *SD* = 6.1 years old)	130	Whole brain	2 × 2 × 2 mm	None	3 T Siemens Magnetom Trio Tim	Mask from Tyszka and Pauli (2016) amygdala sub‐regions atlas. Specially adapted (see supplementary methods in paper) and 25% thresholded	Probabilistic mask developed by Theiss et al. (2017). Thresholded at 25%	Voxel‐wise seed‐based correlation analysis
Hofmann and Straube (2019)	Healthy unrelated young adults from the Human Connectome Project (*M* = 28, *SD* = 3.6, years old)	391	Whole brain	2 × 2 × 2 mm	None	3 T Skyra Siemens	N/A	Probabilistic mask from Torrisi et al., 2015. Thresholded at 20%	Dynamic causal modelling
Torrisi et al. (2019)	Healthy adult volunteers (*n* = 30, 19 of whom from previous Torrisi et al.'s (2015) sample) and 30 demographically matched patients with GAD and/or SAD.	60	Partial	1.3 × 1.3 × 1.3 mm	2.6 mm	7 T Siemens Magnetom	Mask from Tyszka and Pauli (2016) amygdala sub‐regions atlas	As prescribed in Torrisi et al. (2015)	Case/control seed‐based correlation analysis
Weis, Huggins, Bennett, Parisi, and Larson (2019)	Healthy young adults (*M* = 22.2, *SD* = 3.62, years old)	57	Partial	0.859 × 0.859 × 1.80 mm	3.6 mm	7 T MR950 General Electric	Mask from Tyszka and Pauli (2016) amygdala sub‐regions atlas.	Probabilistic mask developed by Theiss et al. (2017).	Voxel‐wise seed‐based correlation analysis
This study	Healthy young adults, mostly made up of family groups, from the human connectome project (*M* = 28.8, *SD* = 3.7, years old).	1,071	Whole brain	2 × 2 × 2 mm	None	3 T Skyra Siemens	Mask from Tyszka and Pauli (2016) amygdala sub‐regions atlas. Same version as Tillman et al. (2018)	Probabilistic mask developed by Theiss et al. (2017). Thresholded at 25%	Voxel‐wise and targeted region seed‐based correlation analysis

Abbreviations: BST, bed nucleus of the stria terminalis; CeA, central nucleus of the amygdala; ExtA, extended‐amygdala.

Despite some agreement regarding the ExtA ICNs (overlapping connections to medial prefrontal, hippocampal, wider amygdala, and thalamic regions), because of data acquisition, processing differences (such as brain coverage and choice of mask), and repeated use of the same samples, the convergence between studies can be hard to assess (Table 1). Thus, our first aim was to establish the ICNs of the BST and CeA in a large (*n*= > 1,000) independent dataset—the Young Adults Human Connectome Project (HCP). A major strength of this approach is our use of the HCP data. The HCP contains high‐quality imaging data, with most participants having undergone an hour of tf‐fMRI (Glasser et al., 2013, 2016). Scan lengths longer than 10 min are important as studies have highlighted the negative effects of short scan times on the stability of brain function estimates (Birn et al., 2013; Elliott et al., 2020). There is presently some debate as to whether the ExtA acts mostly as a unified structure, or whether its components represent separate systems underlying different processes, in particular with regard to fear versus anxiety processing or in the tracking of threat imminence (Fox & Shackman, 2019; Goode et al., 2019, 2020; Hur et al., 2020; Tillman et al., 2018; Walker, Miles, & Davis, 2009). Therefore, we utilised this sample to examine the degree of overlap between the ICNs of the CeA and BST; giving an indirect indication as to the similarity of their functions (Gorka et al., 2018; Oler et al., 2012; Tillman et al., 2018; Torrisi et al., 2015, 2019; Weis et al., 2019).

While phenotypes such as anxiety, fear, depression, and substance use are often studied as if they were separate constructs, they are frequently highly comorbid and demonstrate an overlap of symptoms (Hur, Stockbridge, Fox, & Shackman, 2019; Plana‐Ripoll et al., 2019). Recent work has suggested that these phenotypes can be represented by broader overarching constructs, conceptualised as “dispositional negativity” or simply “negative affect” (Hur et al., 2019; Krueger et al., 2018; Shackman et al., 2018; Shackman, Stockbridge, et al., 2016; Shackman, Tromp, et al., 2016; Waszczuk et al., 2020). Genetic correlation studies have lent credence to this hypothesis, demonstrating that many phenotypically similar traits such as anxiety and depression also share a large proportion of underlying genetic risk factors (Allegrini et al., 2020; Hur et al., 2019; Waszczuk et al., 2020). Human and non‐human primate neuroimaging work suggests that dispositional negativity traits are associated with networks that include the ExtA, with a particular focus on the central amygdala (Hur et al., 2019). Consequently, to expand on this previous work, we placed self‐report questionnaire measures examining phenotypes of interest (anxiety, depression, fear, and alcohol use) into a principal component analysis (PCA). We then used these principal components to test for associations with the ExtA ICNs. Human studies examining self‐report trait associations with ExtA ICNs have so far been limited by small sample sizes, which hinder the power to detect an effect. Here, we addressed this issue by using a large population‐level sample containing multiple measures of relevant phenotypes.

Psychological traits and aspects of brain function, such as iFC, can be partly attributed to genetics (Adhikari et al., 2018; Colclough et al., 2017; Elliott et al., 2018; Elliott et al., 2019; Yang et al., 2016). Because psychological traits are underpinned by the brain, understanding whether psychological traits and brain function share underlying genetic influences can be useful for identifying where research may be able to detect biological mechanisms contributing to both. Despite its apparent importance in a range of psychopathology‐linked behaviours, to our knowledge only one study to date has examined genetic co‐variance of psychopathology‐associated traits with ExtA iFC. This study used a pedigree of rhesus monkeys to demonstrate that iFC between the CeA and an area consistent with the BST was co‐heritable with anxious temperament (pgr = 0.87) (Fox et al., 2018). While heritability estimates do not alone provide information about the nature of shared genetic mechanisms (Turkheimer, 2016), this result suggests that ExtA iFC and anxiety‐related traits may be influenced by common genetic factors.

Therefore, we used the kinship structure of the HCP data to estimate within BST—CeA iFC heritability and co‐heritability with our principal components. Thus, we aimed to extend the non‐human primate finding of Fox et al. to humans by demonstrating that within BST‐CeA iFC is both heritable and co‐heritable with anxiety‐related traits (Fox et al., 2018). Previous evidence has also reported significant BST iFC to other amygdala sub‐nuclei in humans (Hofmann & Straube, 2019). Hence, we further ran a post hoc analysis to assess the heritability and co‐heritability (with the principle components) of BST iFC to the centromedial, basolateral, and superficial amygdala regions.

## 2 METHODS

### 2.1 Sample descriptions

#### 2.1.1 The Human Connectome Project

Participants were drawn from the April 2018 release of the Young Adults HCP study (*n* = 1,206) (Van Essen et al., 2012). Participants were between the ages of 25–37 and primarily made up of family groups, with an average size of three to four members and most containing a MZ (273) or DZ (166) twin pair. Participants were excluded during initial recruitment for psychiatric, neurological, or other long‐term illnesses, although participants who were overweight, smoked, or had a history of recreational drug use and/or heavy drinking were included (Van Essen et al., 2012). For the imaging analysis, our samples included participants who had at least one tf‐fMRI scan (*n* = 1,096). Of these, there were 596 females and 500 males. For detailed recruitment information and for a full list of procedures see: https://www.humanconnectome.org/study/hcp-young-adult. See the supplementary material for a breakdown of participants demographic information.

### 2.2 Principal component analysis

In this study, phenotypes of interest were those related to anxiety, depression, fear, and substance use. There are multiple instruments in this dataset measuring each of these constructs and these phenotypes are frequently highly correlated. Therefore, we performed PCA and reduced data dimensionality by extracting the minimum number of latent components that summarise the maximum amount of information contained in the original measures. The questionnaire measures outlined in the next section were joined into a single dataset and were tested for sampling adequacy using a Kaiser–Meyer–Olkin (KMO) test (Dziuban and Shirkey, 1974), followed by the Barlett's test of sphericity. The measures were standardised automatically during analysis and missing values were imputed by the mean of the variable (a maximum of 25/1,206 datapoints, see Table 2). Following the PCA, components were selected if they had an eigenvalue greater than 1 (Bourbon‐Teles et al., 2019). The PCA was conducted in R Studio using the software package FactoMineR (Lê et al., 2008).

**TABLE 2:** PCA items

Item	Questionnaire	Description	*N* (/1,206)	Mean	*SD*
DSM_Anxi_Raw	Achenbach self‐report	SUB‐scale reflecting DSM oriented anxiety traits	1,198	3.94	2.70
DSM_Depr_Raw	Achenbach self‐report	SUB‐scale reflecting DSM oriented depression traits	1,198	4.24	3.45
ASR_Anxd_Raw	Achenbach self‐report	SUB‐SCALE REFLECTING “anxious‐depression” (traits empirically derived)	1,198	5.93	5.40
FearSomat_Unadj	NIH fear affect survey	Somatic symptoms related to arousal	1,205	52.03	8.31
Fear_Affect_Unadj	NIH fear affect survey	Self‐reported fear and anxious misery	1,205	50.28	8.08
PercStress_Unadj	Stress and efficacy self‐report	A scale representing how unpredictable, uncontrollable and overloading respondents find their lives	1,205	48.48	9.17
Total drinks 7 days	Alcohol use survey	Self‐reported alcoholic drinks over the last 7 days	1,179	4.75	7.04
SSAGA_Alc_D4_Dp_Sx	Alcohol use survey	DSM4 alcohol dependence criteria count	1,204	0.55	0.84
SSAGA_Alc_D4_Ab_Sx	Alcohol use survey	DSM4 alcohol abuse symptoms count	1,204	0.27	0.58

*Note:* A description of the questionnaire measures that were entered into the PCA analysis. *N* refers to the number of participants who had data for that particular questionnaire.

Abbreviation: PCA, principal component analysis.

#### 2.2.1 Questionnaire selection

The questionnaires used were administered to each participant by the HCP team and all measures were selected from the NIH toolbox, a well‐validated set of metrics for quick assessment of cognitive, emotional, sensory and motor functions (Weintraub et al., 2013). Items were selected if they measured anxiety, stress, fear, or substance use. Where individual items were not provided, we used the relevant questionnaire subscales (Table 2). For the substance use metrics, we only included measures of alcohol use, as self‐reported smoking and “harder” drug use rates were low (<20% for tobacco use, <8% ever used cocaine). In total, nine measures were selected (Table 2).

### 2.3 Image acquisition and pre‐processing

#### 2.3.1 HCP image acquisition

All images were acquired on a 3 Tesla Skyra Siemens system using a 32‐channel head coil, a customised SC72 gradient insert (100 mT/m) and a customised body transmit coil. Tf‐fMRI scans took place over four 15‐min runs, split between two sessions (two runs in each session). Participants were instructed to keep their eyes open with a fixation cross being projected onto a screen with a dark background in front of them. Within each session oblique axial acquisition alternated between phase encoding in a left‐to‐right or right‐to‐left direction. Functional images were acquired using a multiband gradient echo EPI sequence (TR 720 m; TE 33.1 ms; 72 oblique axial slices; FOV 208 × 108 mm^2^; flip angle 52°; matrix 104 × 90; echo spacing 0.58 ms; 1,200 images per run). High resolution anatomical images were also acquired using a 0.7 mm isotropic T1‐weighted 3D magnetisation‐prepared rapid gradient echo sequence (TR 2,400 ms, TE 2.14 ms, FOV 224 × 224 mm^2^, flip angle 8°) (Glasser et al., 2013; Smith et al., 2013).

#### 2.3.2 HCP pre‐processing

We used the minimally processed tf‐fMRI 3 T dataset, described elsewhere (Glasser et al., 2013). Scripts to run the pipeline are freely available online at https://github.com/Washington-University/HCPpipelines. Briefly, the pipeline applies gradient distortion correction to account for spatial distortions, followed by volume realignment to compensate for subject motion, co‐registration of the fMRI data to the structural image, non‐linear registration to MNI space, intensity normalisation to a mean of 10,000, bias field removal, and masking of data with a brain mask. Structured noise was cleaned from the data by combining independent component analysis (ICA) with the automated component classifier tool FIX ICA (Griffanti et al., 2014; Salimi‐Khorshidi et al., 2014). Finally, head motion time series were regressed out using a 24 confound time series containing the 6 rigid body parameter time series, their temporal derivatives as well as the resulting 12 regressors squared (Glasser et al., 2013, summarised by Hofmann and Straube (2019)). This pipeline was optimised for the HCP dataset and had the aim of maximising the reduction of structured noise components, such as those caused by subject motion, while retaining spatially specific bold signal components (i.e., ICNs) (Glasser et al., 2016). This was reportedly achieved with better than 99% accuracy (Glasser et al., 2016; Griffanti et al., 2014). To reduce the effects of signal drop‐out (Schwaferts, 2017), for each participant a single 4D image was created by taking a mean of their scans using the FSLMaths (Jenkinson, Beckmann, Behrens, Woolrich, & Smith, 2012) mean function. To further mitigate against spurious and systematic iFC correlations resulting from subject motion, we included mean frame‐wise displacement (MeanFD) as a covariate in the phenotype and (co)heritability analyses. Participants with a MeanFD of >0.2 mm were excluded from these analyses (*n* = 9) (Power, Barnes, Snyder, Schlaggar, & Petersen, 2012). As a final precautionary check, we ran a correlation between MeanFD and our phenotypes of interest (the principal components and functional connections), which revealed no significant correlations (supplementary material).

### 2.4 Seed‐based correlation analysis

#### 2.4.1 ExtA seed regions

**FIGURE 1:**
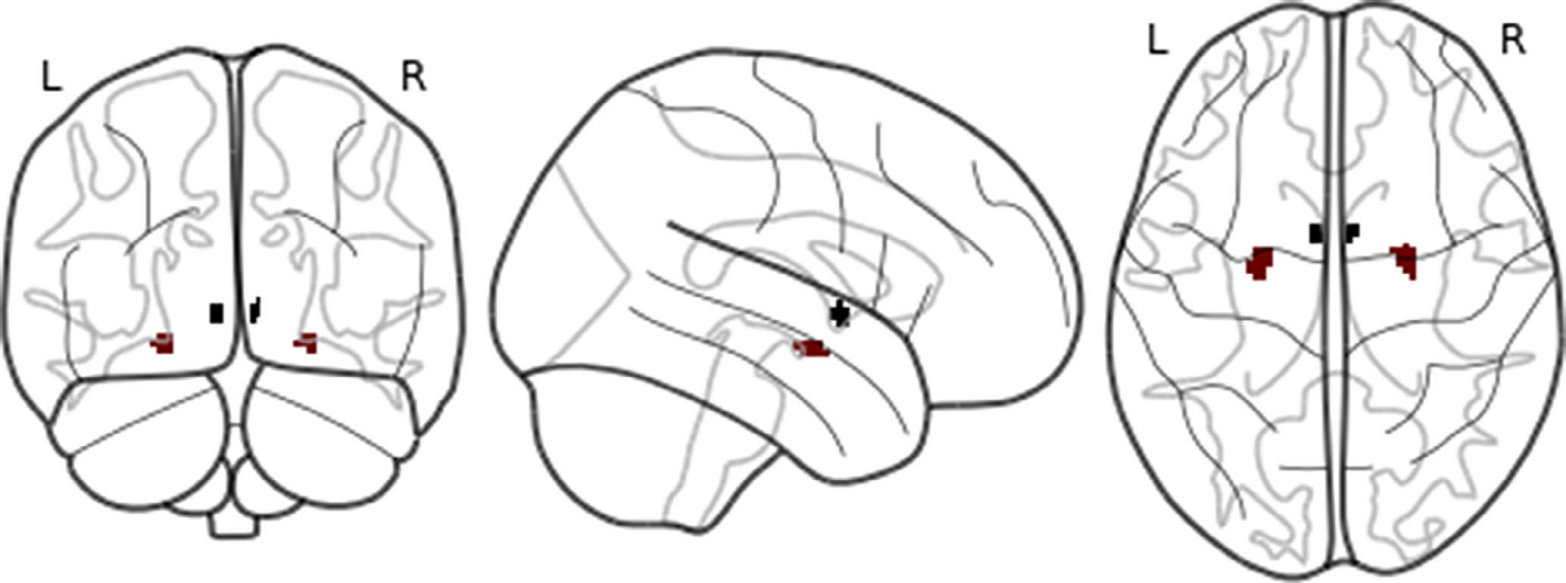
The bed nucleus of the stria terminalis (BST) (blue) and central nucleus of the amygdala (CeA) (red) seeds

**FIGURE 2:**
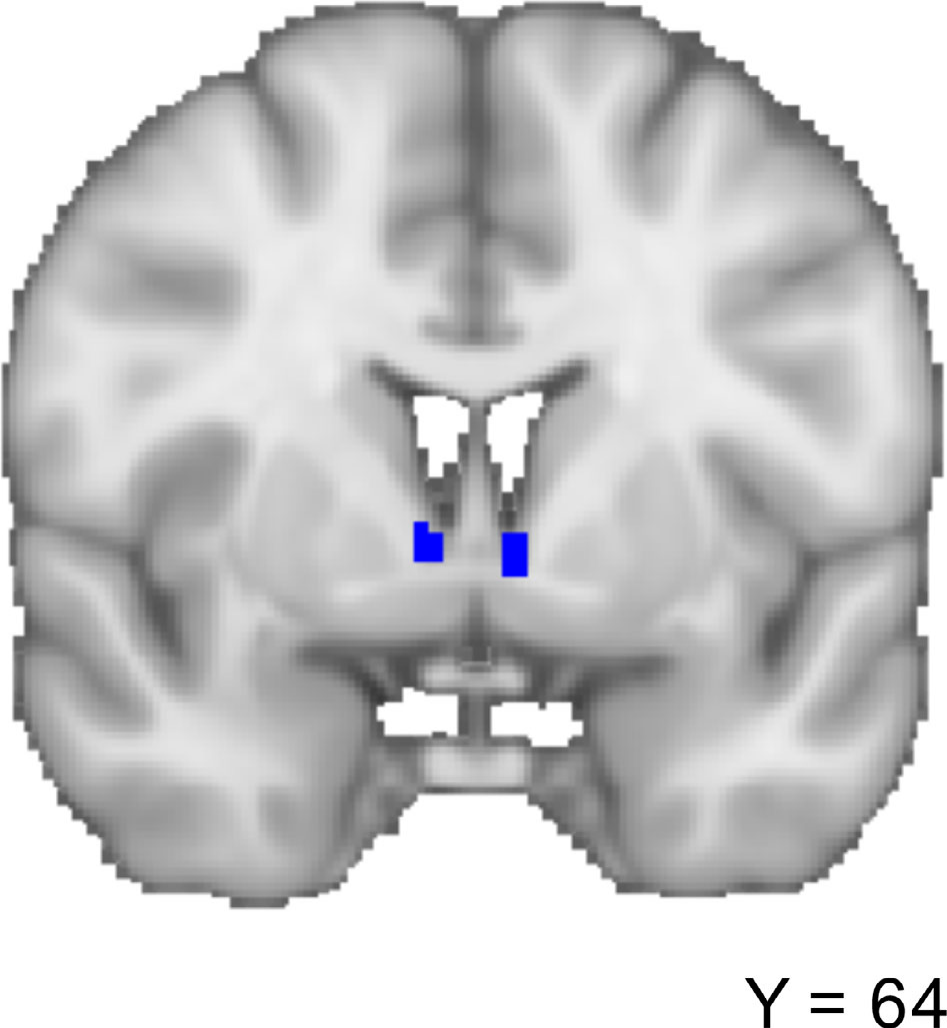
The bed nucleus of the stria terminalis (BST) seed (blue), coronal section

We used two anatomically derived bilateral seed regions for the ExtA, one for the BST and one for the CeA (Figures 1 and 2). The masks were downloaded on March 25, 2019 from a repository on the NeuroVault website (https://neurovault.org/collections/3245) (Tillman et al., 2018). All analyses were run separately for each seed region. Both seeds were thresholded at 25% before use (Tillman et al., 2018) (Figures 1 and 2).

The 3 T 2 mm BST mask was generated by a manual segmentation process undertaken on 10 healthy individuals using a scanning sequence that provided high grey matter/white matter/CSF contrast (Theiss et al., 2017) (Figures 1 and 2). The protocol was found to have high reliability among raters (Dice similarity coefficient ≥ 0.85).

The CeA mask was generated by an experienced neuroanatomist, building on a process developed through a series of studies (Birn et al., 2014; Najafi, Kinnison, & Pessoa, 2017; Oler et al., 2012, 2017; Tillman et al., 2018). Briefly, this was achieved using a specially processed version of the CITI168 high‐resolution (0.7 mm), multimodal (T1/T2) probabilistic template (Tyszka & Pauli, 2016), and was guided by the Mai human brain atlas (Mai, Majtanik, & Paxinos, 2015).

#### 2.4.2 Whole‐brain seed‐based correlation analysis

Seed‐based correlation iFC analysis provides a measure of temporal coherence between a seed‐region's blood‐oxygenation‐level‐dependent (BOLD) activation over time and that of the target regions. Temporal coherence in tf‐fMRI data is used to infer iFC (Battistella et al., 2020; Suárez et al., 2020; Thomas Yeo et al., 2011). To run the analysis we used the *ciftify_seed_corr* tool downloaded from https://edickie.github.io/ciftify/#/ (Dickie et al., 2019), which was in turn adapted from the HCP minimal processing pipeline (Glasser et al., 2013). This works by first extracting a mean time‐series of the seed‐region. This time‐series is then correlated with the mean time‐series of the target regions, producing a Fisher's *r* correlation map. These correlation coefficients are then converted to normally distributed z‐scores using a Fisher r‐z transform (Fisher, 1915). This produces a z‐map for each participant that represents the strength of the correlation of activity for each target region and the seed‐region. We used a whole‐brain voxel‐wise approach, meaning that our target regions were every 2 mm voxel in the brain.

### 2.5 fMRI statistical analysis

#### 2.5.1 Permutation‐based one‐sample *t* tests

Following the creation of a single z‐map for each participant, all of these images were visually inspected. Twenty‐three participants had images removed from further analysis due to having either sections of the signal missing or for having z‐score distributions containing too many values within the outer or inner tail distributions (assessed via fslstats ‐r‐R and histogram plots). The remaining 1,071 participants had their images merged across all participants to create a 4D image using the fslmerge tool (Jenkinson et al., 2012). Permutation‐based one‐sample *t* tests were then run to see which voxels had activity that was significantly correlated with the seed‐regions across all participants. This was done using FSL's PALM command line tool (Winkler et al., 2016).

For the quantification of the whole brain ICNs, we wanted the results to be generalisable to the wider population, thus we were not interested in the influence of family effects across the whole network. Therefore, because our sample was made up of siblings, it was important to account for relatedness such that model estimations were not inflated. PALM permits a kinship matrix that details the family structures within the population. PALM shuffles the data within and between blocks according to this family structure, avoiding relatedness confounding the results. The kinship file was generated with the HCP2Blocks MATLAB script provided online at https://brainder.org/2016/08/01/three-hcp-utilities (Winkler, Webster, Vidaurre, Nichols, & Smith, 2015).

PALM has several optional commands. We used threshold‐free cluster‐enhancement (TFCE) and Gamma approximation. Briefly, TFCE enhances cluster‐like structures in the data without having to define somewhat arbitrary cluster thresholds beforehand (Smith & Nichols, 2009). Gamma approximation is an option used to speed up the analysis by running a smaller number of permutations, computing empirically the moments of the permutation distribution and then fitting a gamma distribution (Winkler et al., 2016). The number of permutations used was 1,000.

#### 2.5.2 Post hoc thresholding of PALM output images

Given the large sample size, the vast majority of voxels in the brain were statistically significantly correlated to our seed‐regions after family wise error rate correction. To reveal meaningful connections and to reduce noise, we further thresholded the images post hoc using the t‐statistic. This was done by visually inspecting the output images and choosing a t‐score that met the criteria of delineating meaningful anatomical structures in the brain, while keeping the maximum amount of signal (Tillman et al., 2018). The t‐threshold we used for both seed‐images was 9. Using the ‐saveglm option from PALM, we saw that this equated to a minimum Cohen's *d* value of 0.275 (Winkler et al., 2016). While we are confident this was an appropriate threshold, given the somewhat arbitrary nature of this method, thresholded and un‐thresholded output images have been uploaded to NeuroVault for inspection at https://identifiers.org/neurovault.collection:8076.

#### 2.5.3 Analysing shared and unique BST and CeA networks

To assess the shared ICNs between the BST and CeA, we used a minimum conjunction (Boolean “AND”) to combine the t‐thresholded PALM output images of each seed (Nichols, Brett, Andersson, Wager, & Poline, 2005; Tillman et al., 2018). This created a new image displaying the areas of ICNs that overlapped between the two ExtA regions.

To assess the unique BST and CeA networks, we performed a single group paired difference *t* test using the method outlined on the FSL GLM website (https://fsl.fmrib.ox.ac.uk/fsl/fslwiki/GLM#Single-Group_Paired_Difference_.28Paired_T-Test.29). Briefly, to get the unique BST ICN, we subtracted each participants BST z‐score image from their CeA z‐score image and then ran a one‐sample permutation *t* test on this difference map. This was repeated for the CeA network (CeA—BST z maps, followed by a one‐sample *t* test). A mask was used to restrict analysis to the regions that were found to be connected to one or both seeds in the original one‐sample *t* tests, thus avoiding the need to interpret differences in regions not significantly connected to the seeds (Tillman et al., 2018).

#### 2.5.4 Region identification

Connected regions were identified using a mixture of the Oxford cortical/sub‐cortical atlas and the Juelich Histological Atlas, both provided with FSL (Jenkinson et al., 2012). For iFC to basal ganglia structures and the hypothalamus, we used a collection of masks provided online at Neurovault (https://identifiers.org/neurovault.collection:3145) (Pauli, Nili, & Tyszka, 2018).

### 2.6 Intrinsic connectivity networks and principal component association tests

Following the one‐sample *t* tests for each seed region, we then created a mask of the t‐thresholded significantly connected regions. This mask was then applied to the 4D image of participants connectivity z‐maps to select only the thresholded connected voxels for association testing with our PC's and for gender effects. We used the PALM command‐line tool, with TFCE, Gamma‐approximation, and event blocks to control for family relatedness (see Section 2.5.1). As well as the standard correction for multiple comparisons within each image, PALM further allows for correction across different contrasts with the ‐corr‐con option (Winkler et al., 2016). This option was used along with the ‐demean function, which mean‐centres the variables, and the ‐cmcx function, which allows for synchronised permutations accounting for repeated elements in the design matrix. Three tests were run in total on each seed‐image, one each for the two principal components and one for gender (male, female). Age, age^2^, gender, and MeanFD were used as covariates for all tests, except that gender was of course not included as a covariate for the direct test of gender effects. The number of permutations was 2000 for each test.

### 2.7 Within BST—amygdala heritability, co‐heritability, and phenotype association analysis

**FIGURE 3:**
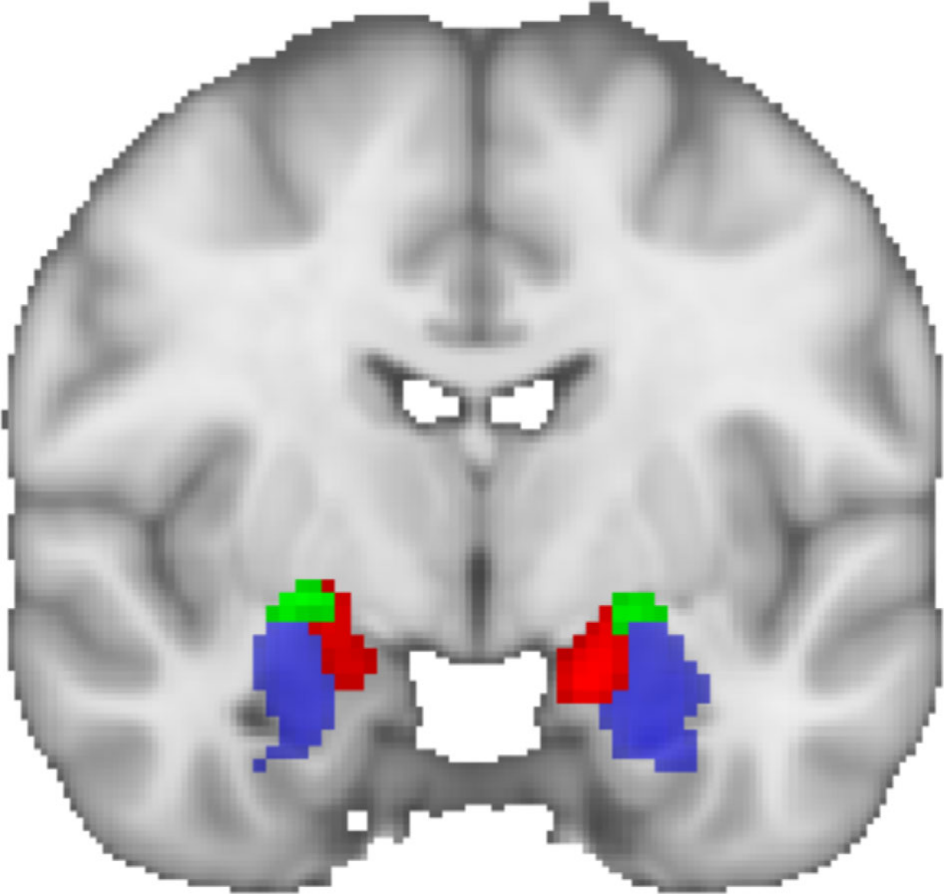
The Juelich Histological Atlas (Eickhoff et al. (2005)) amygdala subregions. Blue = basolateral, green = centromedial, red = superficial. Masks shown were thresholded at 50%

We used the SOLARIUS package for R (Ziyatdinov et al., 2016) to assess the following the (a) heritability of within BST‐CeA iFC; (b) co‐heritability of the within BST‐CeA iFC with each of the two principal components; and (c) phenotypic (rho), genetic (rhog), and environmental (rhoe) correlations between BST‐CeA iFC and each of the two principal components. We further ran a post hoc analysis, conducting the same tests but examining BST iFC with the superficial, centromedial, and basolateral amygdala regions. These regions were defined using the Juelich Histological Atlas, thresholding the probabilistic masks at 50% (Eickhoff et al., 2005) (Figure 3).

SOLARIUS is the R version of the widely used SOLAR‐eclipse software for genetic analysis (Almasy & Blangero, 2010). SOLAR uses a kinship matrix to estimate the proportion of variance in a phenotype attributable to additive genetics, the environment, or to residual error. In this case, we were only permitted to calculate the additive genetic component, as to partition environmental and error effects you require household information that is not provided by the HCP. In this model, monozygotic twins are given a score of 1 and dizygotic twins /siblings of 0.5 to indicate the estimated proportion of shared genetic variation. Half‐siblings were excluded from the analysis (*n* = 88). The pedigree file was created using the HCP2Solar MATLAB function, a tool specifically designed for the HCP participants (https://brainder.org/2016/08/01/three-hcp-utilities) (Winkler et al., 2015). Because the model is sensitive to kurtosis, the phenotype values were inverse normally transformed. SOLARIUS allows analysis of co‐heritability by computing bi‐variate genetic correlations (Kochunov et al., 2019). During the analysis, SOLARIUS computes an estimate of phenotypic, genetic, and environmental correlation between the variables, which we used to assess the relationships between the clusters iFC and component scores. Participants were excluded if they had a MeanFD >0.2 mm (*N* = 9). The covariates for all analyses were MeanFD, sex, age, age^2^, sex × age, and sex × age^2^. The final number of participants in these analyses was *n* = 933. For discussion on using SOLAR for genetic neuroimaging, see Kochunov et al. (2019).

## 3 RESULTS

### 3.1 BST and CeA intrinsic functional connectivity networks

All connected regions described below are the regions visible after the thresholding at *t* = > 9. Negative correlations were observed only within small regions surrounding the ventricles or white matter, and are not reported here. See Tables 3 and 4 for significantly connected clusters with more than 10 voxels. Interactive 3D images of the results have been uploaded to NeuroVault at https://identifiers.org/neurovault.collection:8076.

**TABLE 3:** : Significantly connected clusters to the BST

Cluster index	Voxels	Max t	X	Y	Z	Hemisphere	Region(s) in cluster
271^a^	6,135	16.6	45.8	30	46.2	B	Precuneus cortex, lateral occipital cortex, occipital pole, posterior cingulate gyrus, intracalcarine cortex, middle/superior temporal gyrus, angular gyrus, left hippocampus dentate gyrus, left hippocampus subiculum, left hippocampus cornu ammonis, lingual gyrus, ventral posterior thalamus
270	3,419	15.3	47.5	53.6	61.5	B	Post‐central gyrus, pre‐central gyrus, primary somatosensory cortex, pre‐motor cortex, primary motor cortex, inferior‐frontal gyrus, Broca's area, anterior cingulate gyrus
269	665	14.8	70.9	53.4	41.5	L	Central opercular cortex, primary auditory cortex, insular cortex
268	565	15.2	18.5	54.7	41.9	R	Central opercular cortex, primary auditory cortex, insular cortex
267	279	13.1	45.2	91.8	39.3	B	Frontal pole, paracingulate gyrus, frontal medial cortex
266	138	11.9	33.3	24.9	28.2	R	Occipital fusiform, lingual gyrus
265	136	13	56.5	77.2	59.7	L	Middle frontal gyrus, superior frontal gyrus
264	86	12.1	64.6	62.7	65.2	L	Pre‐central gyrus, middle frontal gyrus, pre‐motor cortex BA6L
263	50	10.7	20.1	74	49.8	R	Middle frontal gyrus, Broca's area BA45, inferior frontal gyrus
262	43	12.2	32.5	77.2	59.9	R	Superior frontal gyrus, middle frontal gyrus
261	36	15.4	32.5	54.2	27.9	R	Hippocampus cornu ammonis, hippocampus dentate gyrus, hippocampus subiculum, posterior amygdala
260	32	11.2	27.7	80.7	30.3	R	Frontal pole, frontal orbital cortex
259	24	10.6	24.5	36.8	25.2	R	Temporal occipital fusiform cortex
258	22	11.5	70.2	29.4	30.1	L	Lateral occipital cortex inferior division
257	21	11.4	63	79.6	29.9	L	Frontal orbital cortex
256	21	12	35.9	62.9	29.3	R	Amygdala superficial group
255	19	10.2	62.8	17.8	38	L	Visual cortex V3VL, visual cortex V4
254	19	11.9	53.8	62.2	29	L	Amygdala superficial group
253	16	12.4	62.3	65.6	27.3	L	Insular cortex (anterior, ventral regions)
252	14	11.5	28.2	66.7	27.2	R	Insular cortex (anterior, ventral regions)
251	14	10	29.6	35.2	66.4	R	Superior parietal lobule 7AR
250	14	10.9	32.1	72.6	58	R	Middle frontal gyrus
249	13	10.5	57.2	16	44.6	L	Occipital pole, visual cortex V2 BA18L, visual cortex V3VL
248	13	10.1	52.3	26.1	63.9	L	Superior parietal lobule 7P
247	12	11.3	40.7	88	31.3	R	Frontal medial cortex, frontal pole
246	12	47.9	41.1	63.1	35.8	R	Thalamus (anterior)
245	12	10	24.9	20.1	42.9	R	Lateral occipital cortex superior division
244	12	10.6	68.4	22.7	38.8	L	Lateral occipital cortex inferior division
243	11	16.8	41.6	66.9	37.4	R	Caudate (posterior)
242	10	10.7	61.9	33.7	65.2	L	Lateral occipital cortex superior division, superior‐parietal lobule 7AL
241	10	10.7	61.2	35	26.4	L	Temporal occipital fusiform cortex

*Note:* Significantly connected clusters to the BST following the one‐sample permutation test. Images were thresholded at *t*= > 9 before clusters were identified. Brain regions were listed if they had >50% chance of being within a cluster. Max *t* is the maximum t‐stat located within a cluster. X, Y, and Z columns represent the location of the centre of gravity for the cluster. Hemi indicates the hemisphere in which the cluster resides where B = bilateral, R = right, and L = Left. For ease of interpretation, clusters shown are those with a minimum of 10 connected voxels.

Abbreviation: BST, bed nucleus of the stria terminalis.

^a^ The large 271 cluster may better be reflected as two clusters, one within the occipital/parietal cortex and the other covering the left hippocampal regions seen in cluster 261.

**TABLE 4:** : Significantly connected clusters to the CeA

Cluster index	Voxels	Max t	X	Y	Z	Hemisphere	Region(s) in cluster
101	1,303	20.3	71	55.9	52.3	L	Somatosensory cortex BA1/BA3b, primary motor cortex BA4a, premotor cortex BA6, planum temporale, central opercular cortex, pre‐central gyrus, temporal pole, primary auditory cortex, dorsal posterior insular
100	1,141	19.9	18.9	56.6	54.1	R	Somatosensory cortex OP4/BA3b/BA1, primary motor cortex BA4p, planum temporale, central opercular cortex, pre‐central gyrus, primary auditory cortex
99	831	18	72.8	44.4	38.8	L	Superior temporal gyrus anterior and posterior division, temporal pole, lateral occipital cortex superior division, supramarginal gyrus posterior division, angular gyrus, inferior parietal lobule
98	803	17.7	16.8	48.7	36.5	R	Superior temporal gyrus anterior and posterior division, temporal pole, middle temporal gyrus posterior division, supramarginal gyrus posterior division, angular gyrus, lateral occipital cortex superior and inferior division
97	318	14.9	45.8	91.7	49.2	B	Frontal pole (dorsal), superior frontal gyrus (anterior)
96	263	46.5	32.8	60	28.5	R	Insular cortex, superficial amygdala, temporal pole, laterobasal amygdala, hippocampus cornu ammonis, hippocampus dentate gyrus, sublenticular extended amygdala
95	221	47.4	56.4	59.5	28.3	L	Insular cortex, superficial amygdala, temporal pole, laterobasal amygdala, hippocampus cornu ammonis, hippocampus dentate gyrus, sublenticular extended amygdala
94	174	17.1	44.9	91	31.7	B	Frontal medial cortex, frontal pole
93	145	15.5	44.9	33.8	51.7	B	Precuneus cortex, posterior cingulate gyrus
92	57	15.6	64.8	76.9	29	L	Frontal orbital cortex, dorsal temporal pole
91	41	13.7	24.5	48.2	45.9	R	Parietal operculum cortex, inferior parietal lobule PFcm
90	35	17.6	45	65.5	29.5	B	Posterior subcallosal cortex
89	33	15	26	58.9	43.9	R	Insular cortex (dorsal, posterior), central opercular (posterior)
88	32	12.9	66.1	45	45.1	L	Parietal operculum cortex, planum temporale, primary auditory cortex
87	31	14.8	26.5	80.2	30	R	Frontal pole (ventral), frontal orbital cortex (anterior)
86	16	12.2	46	50.1	64.4	L	Primary motor cortex BA4a
85	14	12.5	29.6	22.6	18.6	R	Cerebellum horizontal fissure

*Note:* Significantly connected clusters with the CeA following the one‐sample permutation test. Images were thresholded at *t*= > 9 before clusters were identified. Brain regions were listed if they had = > 50% chance of being within a cluster. Max t is the maximum t‐stat located within a cluster. X, Y, and Z columns represent the location of the centre of gravity for the cluster. Hemi indicates the hemisphere in which the cluster resides where B = bilateral, R = right, and L = Left. For ease of interpretation clusters shown are those with a minimum of 10 connected voxels.

Abbreviation: CeA, central nucleus of the amygdala.

#### 3.1.1 Shared BST and CeA intrinsic functional connectivity

**FIGURE 4:**
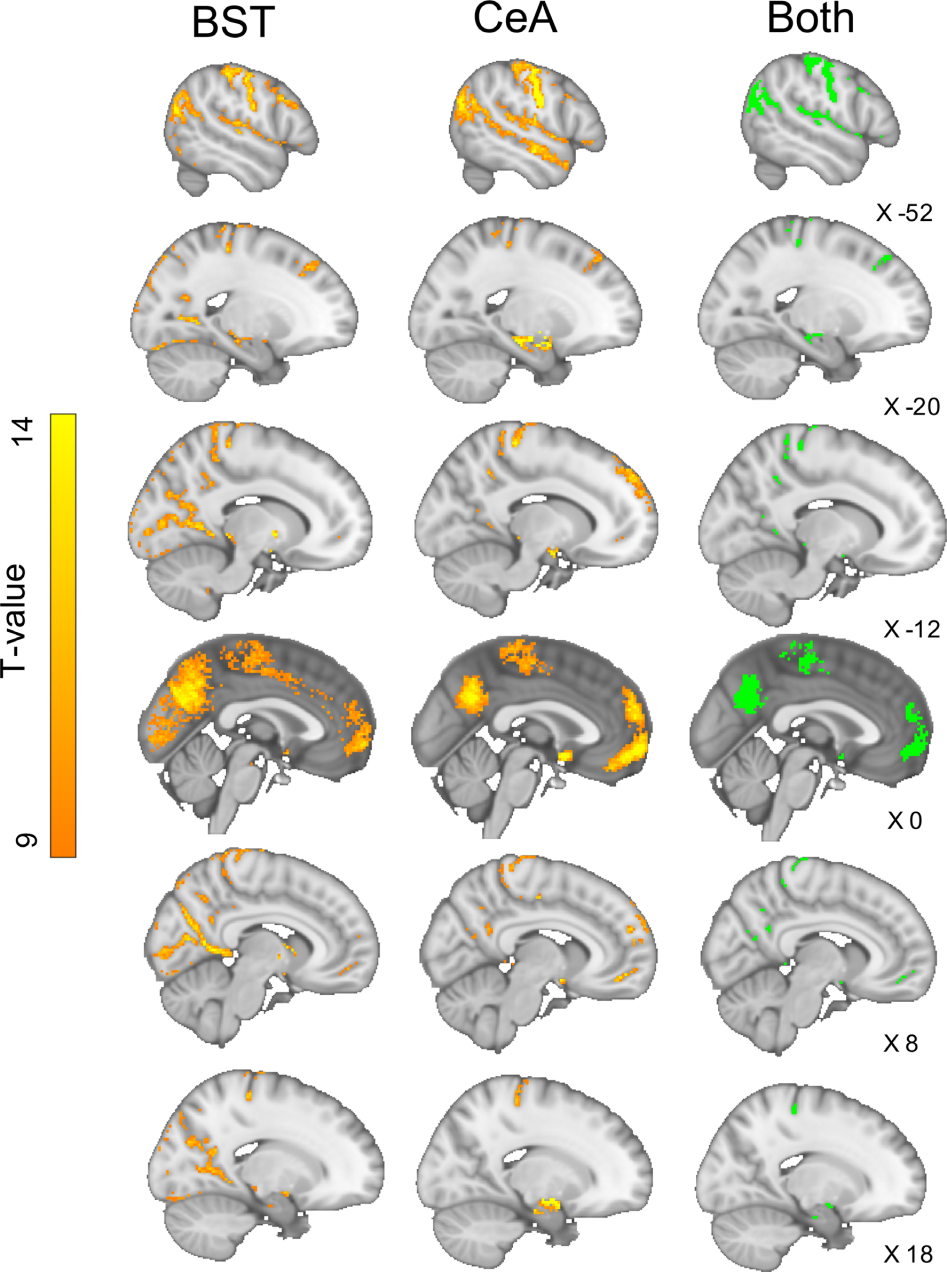
The bed nucleus of the stria terminalis (BST) and central nucleus of the amygdala (CeA) share a common intrinsic functional connectivity pattern, in particular with pre‐frontal cortex, amygdala, hippocampus, superior temporal sulcus, insula, and precuneus. They also share connectivity with areas of the motor and sensory cortex

**FIGURE 5:**
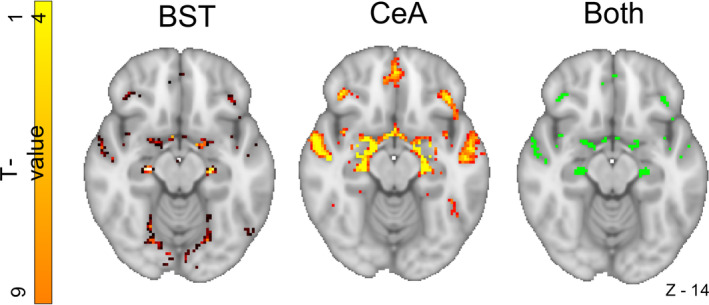
Axial section demonstrating shared connectivity of bed nucleus of the stria terminalis (BST) and central nucleus of the amygdala (CeA) with the hippocampus, insular, temporal gyri, frontal orbital and medial prefrontal cortex. The CeA has more extensive connectivity generally with each of these regions and of note displays unique connectivity along amygdalo‐hippocampal regions

**FIGURE 6:**
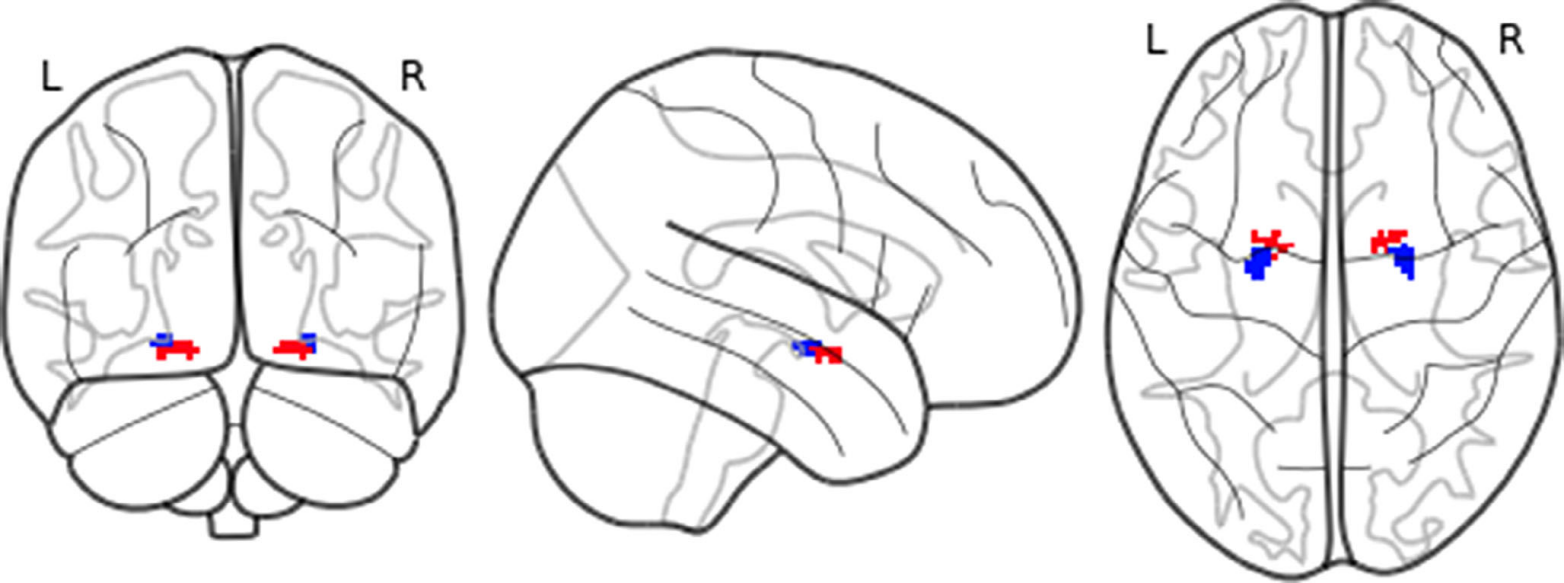
The bed nucleus of the stria terminalis (BST)‐correlated amygdala cluster (red) and central nucleus of the amygdala (CeA) seed (blue)

**FIGURE 7:**
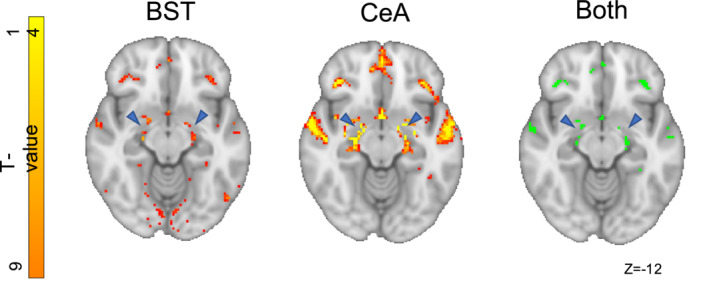
Clusters of connectivity in the region of the sublenticular extended amygdala (SLEA) (blue arrows). This pattern of activity is similar to that reported by Tillman et al. (2018) (Figure 3)

Both the BST and CeA showed significant connectivity with areas including the bilateral hippocampus, superficial amygdala, anterior and posterior‐dorsal insula, frontal orbital cortex, medial prefrontal cortex, frontal pole, anterior paracingulate gyrus, superior temporal gyrus, central opercular cortex, precuneus cortex, and the hypothalamus (Figures 4 and 5, right). There was further shared iFC with pre‐ and post‐central gyri, extending bilaterally to primary motor and sensory regions, and shared connectivity with the angular gyrus/superior lateral occipital cortex. There were no significant voxels directly within either the BST or CeA masks, suggesting that the two regions were not co‐activated at rest. There was however a bilaterally BST‐connected amygdala cluster directly adjacent (within a single voxel) to the CeA mask (Figure 6). The bilateral SLEA region connecting the BST and CeA also demonstrated overlapping connectivity (Figure 7).

#### 3.1.2 BST > CeA connectivity

**FIGURE 8:**
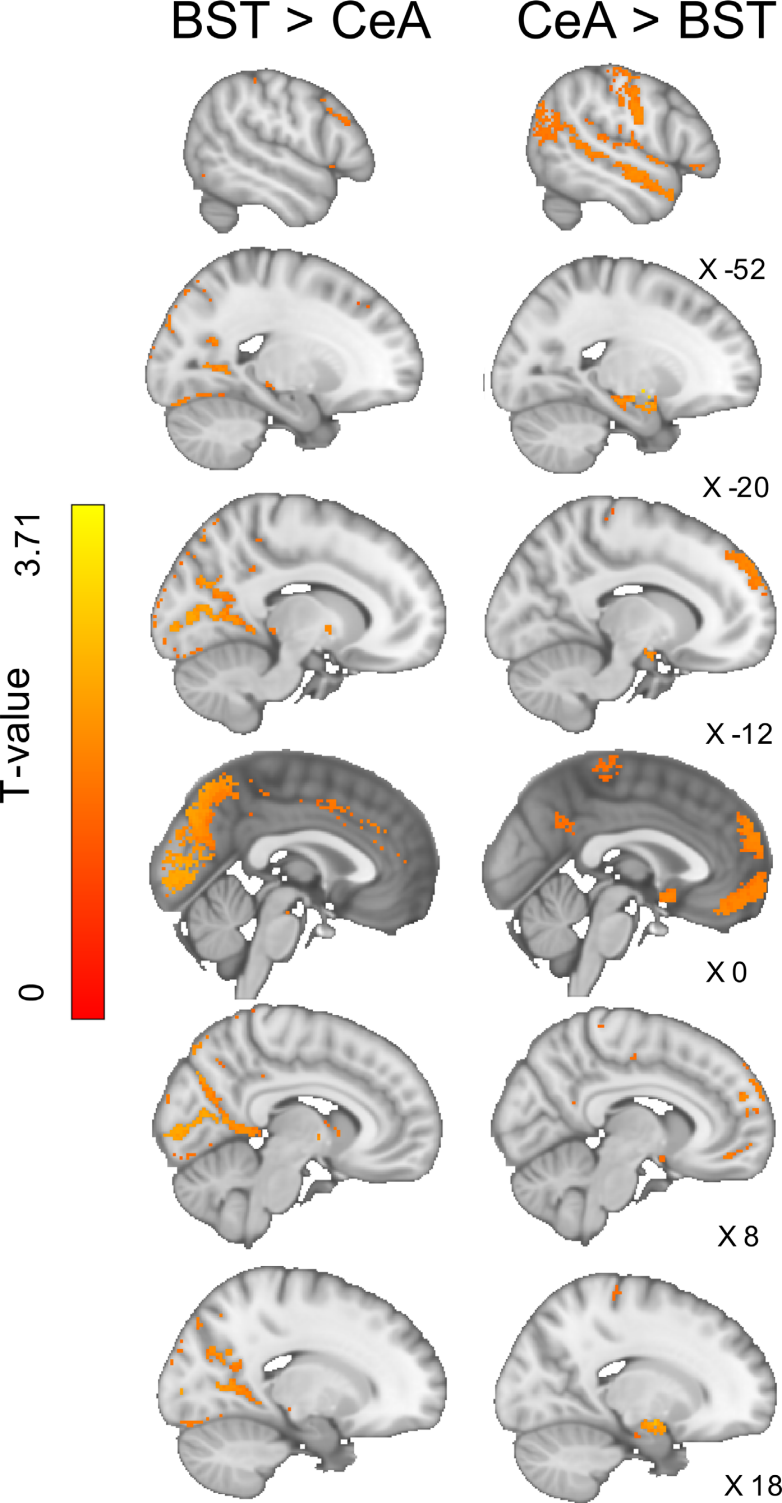
Results of the single group paired‐difference *t* test, showing the unique intrinsic functional connectivity (iFC) to the bed nucleus of the stria terminalis (BST) or central nucleus of the amygdala (CeA) seeds. The BST has greater connectivity with lateral occipital regions and paracingulate gyrus, whereas the CeA has stronger connectivity with the surrounding amygdala, dm‐PFC, temporal poles, and the anterior and superior temporal gyri

The BST had more extensive iFC with the occipital lobe, in particular within the superior occipital cortex, the intracalcarine cortex, and at the occipital pole (Figures 4 and 8, left). There was also greater BST iFC with the posterior and anterior cingulate gyrus, posterior thalamus, precuneus cortex, left and right caudate, globus pallidus, lateral superior frontal gyrus, paracingulate gyrus, and ventral tegmental area (Figures 4 and 8, left).

#### 3.1.3 CeA > BST connectivity

The CeA had greater iFC with the dorsal medial pre‐frontal cortex, frontal pole, temporal pole, central insular, anterior and superior temporal gyrus, supramarginal gyrus, mid‐line superior frontal gyrus, subcallosal cortex, and lateral globus pallidus (Figure 4, middle; Figure 8 right). There was also greater iFC around the surrounding amygdaloid areas (Figure 5, middle) and more extensive connectivity within the SLEA and amygdalo‐hippocampal regions (Figure 6, middle).

### 3.2 PCA results

**FIGURE 9:**
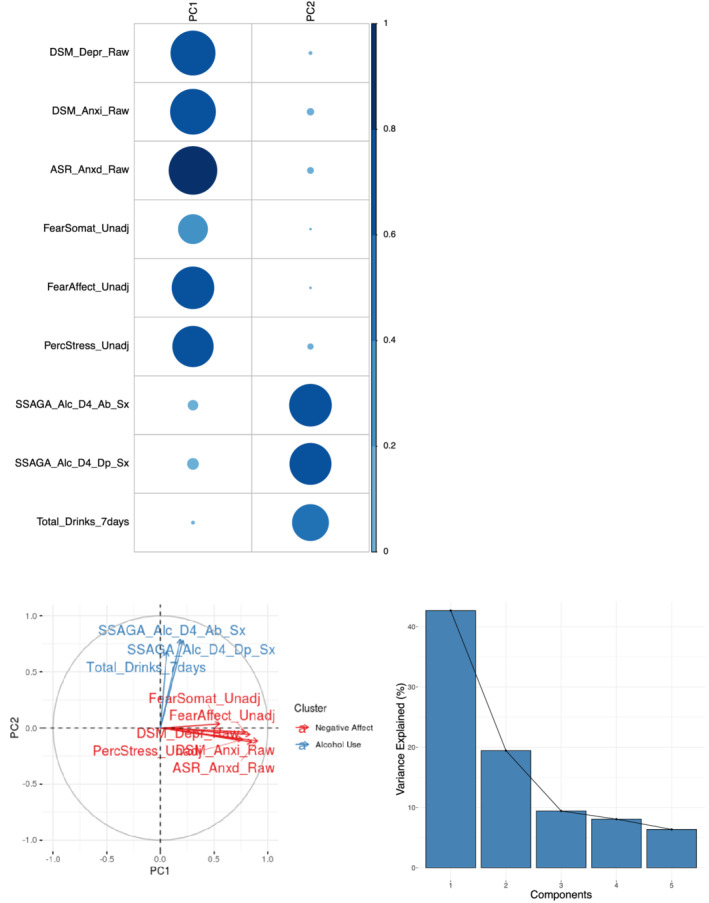
Principal component analysis (PCA) plots. Top left: The circles represent the strength of contribution (cos2) of each questionnaire measure to the principal component. Most measures are represented well by the two principal components (co2 > 5), with FearSomat and Total Drinks 7Days being the least well represented. Bottom left: The correlation circle shows positively correlated variables as being grouped together. Negatively correlated variables are positioned on opposite sides of the plot. Variables that are away from the centre are well represented by that component. Here, it is shown that we can neatly cluster two separate components, representing negative disposition (PC1) or alcohol use (PC2). Bottom right: The screen plot displays the amount of variance explained by each component. The first two components capture 62% of the total variance of the original questionnaire measures. See Table 2 for a description of questionnaire measures

The selected questionnaire items (Table 2) passed the KMO test (overall MSA = 0.8) and Bartlett's test of sphericity (χ^2^(36) = 5,103.77, *p* < .001) indicating that the data was appropriate for PCA. PCA revealed two components with eigenvalues greater than 1 (3.84 and 1.75). These components together explained 62.12% of variability in the data (Figure 9, bottom right). The first component loaded positively on measures capturing negative disposition, such as anxiety, depression and perceived stress, and was therefore named the “negative disposition” component. The second component had significant loadings from alcohol measures and was therefore labelled the “alcohol use” component. See Table 5 and Figure 9 for a breakdown of the PCA results.

**TABLE 5:** Principal component loadings

Item	Dim.1 (cos2)	Dim.2 (cos2)
DSM_Depr_Raw	**0.700**	0.003
DSM_Anxi_Raw	**0.724**	0.016
ASR_Anxd_Raw	**0.819**	0.013
FearSomat_Unadj	0.304	0.001
FearAffect_Unadj	**0.626**	0.001
PercStress_Unadj	**0.587**	0.011
SSAGA_Alc_D4_Ab_Sx	0.035	**0.628**
SSAGA_Alc_D4_Dp_Sx	0.044	**0.608**
Total_Drinks_7days	0.003	0.467

*Note:* This table shows the contribution of each variable to the two principal components (cos2). Highlighted are the items that have a cos2 of .5 and above.

### 3.3 ExtA intrinsic connectivity networks and principal component associations

#### 3.3.1 Intrinsic functional connectivity networks and principal components

The PALM corr‐con analysis provided no evidence that the negative disposition or alcohol use components were significantly associated with increased or decreased iFC across the ExtA ICNs in our sample. Gender was also not associated with the BST or CeA ICNs after correction for multiple comparisons.

### 3.4 Within BST—amygdala iFC heritability analysis

#### 3.4.1 Univariate heritability analysis

Twin‐based heritability analysis of within BST—CeA iFC found no evidence for heritability (Table 6). Analysis of within BST‐centromedial iFC found that this connection was significantly heritable at H2r = 0.15 (Table 6). BST‐superficial iFC had a heritability estimate of H2r = 0.14, but was marginally outside the bounds of statistical significance after FDR correction (Table 6). BST‐basolateral iFC showed no evidence of significant heritability (Table 6). PC1 (negative disposition) was significantly heritable at H2r = 0.22, and PC2 was significantly heritable at H2r = 0.23 (Table 6). Age^2^ was a significant co‐variate for the negative disposition PC; however, it only explained a small amount of variance (0.009). Sex was a significant covariate for the alcohol use PC, with being male demonstrating a small positive influence on the score (0.01).

**TABLE 6:** Results of the univariate heritability analysis

Phenotype	H2r	H2r *SE*	*p*	FDR‐corrected	Significant covariates
BST—superficial amygdala iFC	0.138	0.079	.035*	**0.052**	None
BST—laterobasal amygdala iFC	0.032	0.076	.334	0.401	None
BST—CeA amygdala iFC	0^a^	NA	.500	0.5	None
BST—centromedial amygdala iFC	0.149	0.077	.021*	0.042*	None
PC1 (negative disposition)	0.218	0.081	.002**	0.006**	Age^2^ (*p* = .02*, variance explained = 0.009)
PC2 (alcohol use)	0.225	0.078	.001**	0.006**	Sex (*p* = .01*, variance explained = 0.016)

*Note:* SOLARIUS heritability analysis revealed BST iFC to the centromedial amygdala region was significantly heritable, with BST iFC to the superficial amygdala moving marginally outside the bounds of statistical significance after FDR correction. Principal components one and two were significantly heritable, with age^2^ and sex explaining a small amount of variance in each, respectively.

Abbreviations: BST, bed nucleus of the stria terminalis; CeA, central nucleus of the amygdala; FDR, false‐discovery rate; iFC, intrinsic functional connectivity.

^a^ BST‐CeA amygdala iFC had only a fractional difference between the sporadic and polygenic model likelihood values; therefore, the heritability estimate was 0.

#### 3.4.2 Bivariate heritability analysis

Co‐heritability analysis did not reveal any significant phenotypic, environmental, or genetic correlations with either of the principal components for any of the amygdala sub‐regions (see supplementary material for bivariate SOLARIUS outputs).

## 4 DISCUSSION

### 4.1 Summary of findings

Using a large young adult human sample, we revealed distinct, but overlapping, ExtA ICNs that are largely consistent with findings from smaller previous human neuroimaging studies (Avery et al., 2014; Gorka et al., 2018; Oler et al., 2012, 2017; Tillman et al., 2018; Torrisi et al., 2015; Weis et al., 2019). Genetic analysis of within BST‐ CeA iFC provided no evidence for a heritable connection. However, post hoc analysis of amygdala sub‐regions revealed evidence for small heritability estimates for BST‐centromedial and superficial regions. PCA reduced scores on nine questionnaire measures of anxiety, fear, depression, and substance use to two components, which we interpret as “negative disposition” and “alcohol use.” Contrary to our hypotheses, we report no evidence for associations of these phenotypes across the ExtA ICNs. We also found no evidence that specific BST iFC to any of the tested amygdala regions were co‐heritable or otherwise correlated with either of the components.

### 4.2 Intrinsic connectivity networks of the ExtA

Our shared ICN results are in broad agreement with the previous literature, specifically demonstrating overlapping connections within a now widely reported ExtA ICN that includes the mPFC, bilateral hippocampus, insular regions, wider amygdala areas, and the precuneus (Avery et al., 2014; Gorka et al., 2018; Pedersen et al., 2020; Tillman et al., 2018; Torrisi et al., 2015, 2019; Weis et al., 2019). We report shared iFC to lateral temporal regions, including the superior and middle temporal gyri and the temporal poles, again largely consistent with previous human iFC results. Whist amygdala structural connections to lateral temporal regions are well characterised (Folloni et al., 2019; Janak & Tye, 2015; Klingler & Gloor, 1960), this is not the case for the BST and it has been suggested that BST‐temporal pole connectivity may even be unique to humans (Avery et al., 2014). We demonstrate shared iFC to areas of the sensory/motor cortex, auditory regions, and to lateral occipital areas, something also reported by Tillman et al. (2018). This largely cortical sensory‐motor connectivity is consistent with the suggestion that the ExtA serves as an integrator of sensory information, which can then prepare the motor and endocrine systems to act according to the emotional salience and threat‐relevance of the stimuli (Ahrens et al., 2018; Fox & Shackman, 2019; Goode & Maren, 2017; Lebow & Chen, 2016). Our finding of iFC with frontal regions, in particular the mPFC, is consistent with non‐human primate neural tracer studies and human structural imaging work demonstrating direct structural connectivity with both the amygdala and BST (Chiba, Kayahara, & Nakano, 2001; Crawford, Muhlert, MacDonald, & Lawrence, 2020; Folloni et al., 2019; Krüger, Shiozawa, Kreifelts, Scheffler, & Ethofer, 2015); a finding coherent with theories of emotion regulation (e.g., Banks, Eddy, Angstadt, Nathan, & Phan, 2007; Fox et al., 2010).

For the BST, we report a unique cluster of iFC within visual areas (including V1, V2, and the occipital fusiform gyrus), the posterior thalamus, and the posterior cingulate gyrus. Although BST‐occipital connectivity is not commonly reported in human or pre‐clinical research (McDonald, 1998), a similar pattern was revealed by Tillman et al. (2018), who demonstrated a remarkably similar cluster of iFC in humans stretching from the posterior thalamus, through the lingual gyrus and into the visual cortices. Additionally, a recent study comparing patients with anxiety disorder to controls also reported an unexpected coupling of these two regions, suggesting that abnormal coupling of the BST to the occipital cortex could reflect differences in anxiety‐based interpretation of, or attention to, visual stimuli (Torrisi et al., 2019). Our finding of BST connectivity with areas of the basal ganglia and VTA has been widely reported in human imaging and pre‐clinical neuronal tracer work, whereas iFC with the paracingulate gyrus is only reported in the human literature ( Avery et al., 2014; Gorka et al., 2018; Tillman et al., 2018; Torrisi et al., 2015; Weis et al., 2019). Diffusion tensor imaging by Avery et al. suggested that the human BST and paracingulate are not structurally connected, indicating an indirect functional connection mediated through other structures (Avery et al., 2014).

The CeA exhibited a large cluster of iFC within the mPFC, commensurate with pre‐clinical tracer and human neuroimaging research demonstrating widespread reciprocal structural connections between the amygdala and pre‐frontal regions (Aggleton, Wright, Rosene, & Saunders, 2015; Chiba et al., 2001; Folloni et al., 2019). Temporal lobe connectivity was more robust for the CeA than the BST, reaching deeper into the brain to the mid‐insular and extending further out to an area of the superior temporal regions to the end of the bilateral temporal poles. Extensive amygdala connectivity to the insular and lateral temporal regions has been demonstrated in non‐human primate research as well as in human FC and diffusion MRI studies (Folloni et al., 2019; Janak & Tye, 2015; Klingler & Gloor, 1960). Of interest, a recent human tf‐fMRI mapping of iFC in anxiety disorder patients found that CeA connectivity to the superior temporal gyrus was significantly stronger compared to a control group (Torrisi et al., 2019). The CeA demonstrated unique iFC to wider amygdala structures, as well as the amygdalo‐hippocampal regions. Amygdala–hippocampal connections are thought to be key in the processing of emotionally salient events and manipulation of memory under stress, with the CeA in particular implicated in context‐dependent retrieval of cued fear memories (de Voogd, Klumpers, Fernández, & Hermans, 2017; Sylvester et al., 2020; Xu et al., 2016). Because we only measure correlated BOLD activity, without taking into account more elaborate models that assess causality, we are not permitted to make inferences regarding the direction of connectivity (Rogers, Morgan, Newton, & Gore, 2007). However, an extensive body of work on the amygdala suggests that many of the CeA connections are mediated through the basolateral amygdala to the CeA, which in turn serves primarily as an output to basal forebrain structures (Janak & Tye, 2015). The picture is complex, however, and many studies have also shown direct structural connections with the CeA region, for example, from agranular and dysgranular regions of the insular in Macaques and from the ventral hippocampus in mice (Stefanacci & Amaral, 2002; Xu et al., 2016).

Given pre‐clinical and human imaging results demonstrating structural and functional connectivity between the CeA and BST (Avery et al., 2014; Davis, Walker, Miles, & Grillon, 2010; Fox et al., 2018; Gorka et al., 2018; Hofmann & Straube, 2019; Martin et al., 1991; Oler et al., 2017; Torrisi et al., 2015), we expected to find evidence of strong iFC between our BST and CeA masks, however this was not quite the case. After thresholding, we did not find evidence of CeA iFC with the BST, although we did find a bilateral BST‐functionally connected region directly adjacent to the original CeA mask (Figure 6). Given the small size of the structures, many studies refer to “areas consistent with” the BST and CeA (Fox & Shackman, 2019). These discrepancies can likely be explained by the difficultly of accurately delineating the amygdala sub‐regions using MRI and/or the noisy nature of tf‐fMRI data (Kedo et al., 2018; Sylvester et al., 2020).

Our results revealed minimal connectivity to the thalamus. Given thalamic connectivity is widely reported in structural and functional studies in both pre‐clinical and human studies (Fox et al., 2015; Fox & Shackman, 2019; Lebow & Chen, 2016), it seems likely that this may be due to a difference in data acquisition or pre‐processing. Although speculative, the discrepancy could perhaps be explained by signal drop‐out, something that has been shown to affect FC estimates of the thalamus in the HCP data (Schwaferts, 2017).

In general, though, our findings are highly consistent with the smaller previous studies, and in particular are similar to those of Tillman et al. who, in a different sample, used the same BST and CeA masks (Tillman et al., 2018). While needing to be formally evaluated, this similar pattern of results across samples suggests the existence of a reliable ExtA ICN in healthy humans. If validated, this network could be used as a standard to compare against clinical groups; a technique already used with some success for anxiety disorder patients (Pedersen et al., 2020; Torrisi et al., 2019).

### 4.3 Heritability and co‐heritability of within BST‐amygdala iFC

Contrary to recent primate evidence (Fox et al., 2018), we do not report evidence of a heritable functional connection between the BST and CeA. A post hoc analysis did reveal evidence for a small magnitude of heritability between the BST and the centromedial and superficial amygdala regions; however, there was no evidence of iFC co‐heritability with either of the principal components (negative disposition, alcohol use).

Although brain morphology and development are reliably heritable (Jansen, Mous, White, Posthuma, & Polderman, 2015), this is not necessarily the case for iFC where heritability estimates can frequently be zero (Elliott et al., 2018; Jansen et al., 2015). In an analysis of the SNP‐based heritability of various image‐derived phenotypes in the large UK Biobank human sample (*n* = 8,428), Elliot et al. reported that out of 1771 connectivity edges investigated, only 235 showed evidence of significant heritability, and the average H2r of the significant results was around .15 (Elliott et al., 2018). The reasons for low iFC heritability estimates are not well understood but could reflect either comparatively noisy signal or simply the greater context‐dependent variability inherent within fluctuating connections (Cabeza, Stanley, & Moscovitch, 2018). This makes the Fox primate finding of high heritability (.45) all the more interesting, although the usefulness of comparing the strength of heritability estimates across samples is limited as they are highly influenced by their particular environment; something compounded by comparing across species (Turkheimer, 2016). The fact that we found a heritable connection with the centromedial and superficial amygdala, and not specifically the CeA as was reported in Fox et al., may again reflect difficulties in locating small anatomical regions within the amygdala. With this in mind, our finding of H2r results of ~.14, while smaller than the non‐human primate evidence, is not zero and is broadly in line with other estimates of the heritability of iFC findings in humans (Elliott et al., 2018). Further examination in other human samples could perhaps assess whether individualised task‐based, naturalistic fMRI, behaviourally defined (rather than self‐reported) negative disposition phenotypes, and/or the use of clinical groups influences the heritability estimates of ExtA iFC (Finn et al., 2017). Larger twin‐samples with 7 T MRI data and rich phenotyping would also help to resolve issues around the delineation of amygdala sub‐region boundaries while allowing for co‐heritability analysis, which is after all of primary interest given the suggestion of shared genetic mechanisms.

### 4.4 Principal components and ExtA iFC

Our first principal component grouped together questionnaire items that represented aspects of negative disposition (stress, fear, anxiety, depression), supporting previous work (Hur et al., 2019; Krueger et al., 2018; Shackman et al., 2018; Shackman, Stockbridge, et al., 2016; Shackman, Tromp, et al., 2016; Waszczuk et al., 2020). The ExtA is implicated by numerous pre‐clinical and human studies in aspects of negative disposition, in particular in relation to fear and anxiety (Fox & Shackman, 2019; Hur et al., 2019). It is then perhaps surprising that we report no associations with this principal component across the ICNs. On closer inspection of the literature, however, our finding is in keeping with other iFC studies that have used non‐clinical populations (Pedersen et al., 2020; Weis et al., 2019). Weis et al. reported no robust associations within BST, CeA, or BLA iFC with trait anxiety in a sample of healthy undergraduates (Weis et al., 2019). This was also the case in a study by Pederson et al. who, when looking at within ExtA (i.e., BST‐CeA) iFC found no significant associations with trait anxiety or negative affect in a healthy sample (Pedersen et al., 2020).

Studies that do report ExtA associations with negative disposition phenotypes are overwhelmingly conducted either in clinical populations or during task‐based fMRI where state anxiety or fear is induced (Andreatta et al., 2015; Brinkmann et al., 2018; Choi, Padmala, & Pessoa, 2012; Grupe, Oathes, & Nitschke, 2013; Klumpers, Kroes, Baas, & Fernández, 2017; Mobbs et al., 2010; Naaz, Knight, & Depue, 2019; Pedersen et al., 2020; Torrisi et al., 2019). There could be a number of reasons for this discrepancy. It may simply be that in a relatively healthy sample, even with a large number of participants, the variation in trait negative disposition is too small to detect any resting‐state ExtA network associations. Further to this, recent research has suggested that there is a systematic sampling bias whereby more anxious individuals are reluctant to undergo MRI scanning (Charpentier et al., 2020). Second, although the ExtA is implicated in studies that induce state anxiety, the networks involved in this process may be different to those responsible for having high anxiety as a trait. Torrisi et al. have demonstrated that the ExtA ICN regions that differ between anxiety disorder patients and controls are not the same as those recruited during state anxiety induction (Torrisi et al., 2019). Further, when correlating anxiety symptoms in the patient group with iFC, they found no overlap between the specific anxiety symptoms and the regions that differentiated patients from controls. This study, along with other recent findings (Porta‐Casteràs et al., 2020) suggests that clinical diagnoses, specific symptoms, and trait measures may all be underpinned by different networks. It may be the case then that at a neural level there is little continuity between otherwise healthy people with, for example, high anxiety, and clinical populations (Porta‐Casteràs et al., 2020). As such, revealing the networks implicated in clinical disorders may not be as simple as looking at typical trait variation and extrapolating from these findings. As well, there is some evidence to suggest that individual differences are best observed under emotional or cognitive challenge, rather than at rest (Finn et al., 2017; Stewart, Coan, Towers, & Allen, 2014). In any case, despite associations using task‐based, clinical, and pre‐clinical evidence, at present there does not seem to be good evidence that iFC of the ExtA is related to self‐reported negative disposition in non‐clinical human populations.

Likewise, and perhaps for similar reasons, we found no association of ExtA iFC with our second PC, which represented alcohol‐use. Our sample did not consist of many heavy drinkers, with the median drinks consumed per week being just two, which likely reduced our chances of finding an effect. Despite quite a substantial body of pre‐clinical work linking the ExtA to alcohol consumption (Campbell et al., 2019; Centanni, Bedse, Patel, & Winder, 2019; de Guglielmo et al., 2019; Erikson et al., 2018; Harris & Winder, 2018; Kash, 2012; Pleil et al., 2016; Roberto et al., 2020; Volkow et al., 2016), there is very little investigation of the ExtA and alcohol use in humans; with most work tending to focus on the amygdala proper (Hur et al., 2018; Lebow & Chen, 2016). One study that did specifically examine ExtA iFC found that under the influence of alcohol, BST and CeA reactivity to emotional faces was dampened (Hur et al., 2018). Although we did not find evidence of a self‐report alcohol‐use association in our sample, given the importance of understanding alcohol use behaviours and the strength of evidence from the animal literature, ExtA neuroimaging work on the effects of alcohol in humans should remain a priority. Getting participants to drink alcohol (Hur et al., 2018), utilising heavy drinkers, or making use of task‐based fMRI (Finn et al., 2017) could be a more fruitful approach for identifying ExtA‐alcohol associations.

Our estimates of negative disposition and alcohol use heritability were broadly in line, if not slightly smaller, than similar human studies (Han & Adolphs, 2020; Kranzler et al., 2019; Swan, Carmelli, Rosenman, Fabsitz, & Christian, 1990; Zheng, Plomin, & von Stumm, 2016). As mentioned above, however (Section 4.3), direct comparison of the strength of heritability estimates across samples is of limited value, and as such should not be over‐interpreted (Turkheimer, 2016). The covariates sex and age^2^ were statistically significantly associated with alcohol use and negative disposition, respectively. Age^2^ explained only a tiny amount of variance, and so interpretation is limited in this case. The finding that being male is associated with a small increase in alcohol use scores, however, is in line with recent findings of US samples (White et al., 2015).

### 4.5 Limitations

Our study has some limitations. First, our analyses were conducted using 3 T MRI data. Although imaging at this field strength has been found to accurately capture small regions such as the BST (Theiss et al., 2017), higher resolution, and individualised anatomical parcellations, would enable better characterisation of ExtA iFC networks. Additionally, it is the case that even the small BST structure is made up of further sub‐nuclei that may have distinct functions, a point that is difficult to address using human MRI (Fox & Shackman, 2019; Kim et al., 2013). Second, as is the case with all seed‐based correlation analyses, the interpretation of the results is correlational only and mechanistic inferences including the directionality of the connections cannot be inferred (Mohanty et al., 2020; Pearlson, 2017). Third, although we aimed to be consistent with similar tf‐fMRI HCP studies (Hofmann & Straube, 2019), our choice to favour some pre‐processing techniques over others, such as global signal regression, could have impacted our findings (Glasser et al., 2016; Murphy & Fox, 2017). This is unfortunately a limitation upon all fMRI studies until a consensus approach on pre‐processing steps can be reached (Murphy & Fox, 2017). Finally, our questionnaire measures were all self‐report, which can sometimes affect the accuracy of the phenotyping (Rosenman, Tennekoon, & Hill, 2011). This may be a particular problem for self‐reported drinking behaviour as previous studies have shown heavy‐drinking to be underreported (Northcote & Livingston, 2011).

### 4.6 Conclusions and future directions

We used a large sample of high quality tf‐fMRI data to assess the ICNs of the two key ExtA nodes. Our ICN findings largely replicated previous tf‐fMRI mapping work, implicating the nodes in mostly overlapping ICNs that includes iFC with medial pre‐frontal, hippocampal, wider amygdala, lateral temporal, and precuneus regions. Although for our analysis we intended to establish the ExtA ICNs unencumbered by family relatedness, so as to enable inferences to the wider population, future work could intentionally explore how family relatedness influences the networks. This would allow for heritability and co‐heritability analysis across the entire ICNs, instead of a priori selected regions. We report for the first time in humans that within BST‐ centromedial and superficial amygdala iFC is heritable. We did not replicate the recent non‐human primate finding (Fox et al., 2018) of BST‐CeA iFC co‐heritability with an anxiety‐related phenotype. We found no evidence for network associations with negative disposition or alcohol use principal components. Recent work has suggested that self‐report trait effects may not be associated with the same neural networks as those identified under task‐based conditions and in clinical groups. Future work should explore further these differences by using a combination of self‐report, task‐based measures, and clinical groups (e.g., Porta‐Casteràs et al., 2020). Given that this tf‐fMRI network appears to be reliably delineated across healthy samples, researchers should move towards more causal approaches to probe its function. As it has been shown that the ExtA has many functional and structural cortical connections, one approach could be to use brain stimulation techniques to alter the ExtA network via a cortical node to see whether this impacts on related functions. This type of analysis has already been used effectively to probe other subcortical–cortical networks, for example, those involving memory and the hippocampus (e.g., Warren, Hermiller, Nilakantan, & Voss, 2019).

## CONFLICT OF INTEREST

The authors declare no conflict of interest.

## Supporting information


**Appendix** **S1:** Supplementary Information

## Data Availability

All primary data used in this study is available on request from the HCP, which can be found at https://www.humanconnectome.org/study/hcp-young-adult. Output images from this study have been uploaded to NeuroVault at https://identifiers.org/neurovault.collection:8076. The code used to generate the data can be made available upon request to the lead author (berrysc@cardiff.ac.uk).
