# Supplementary material for: Extended‐amygdala intrinsic functional connectivity networks: A population study
Source: Hum Brain Mapp. 2020 Dec 12;42(6):1594–616. doi: 10.1002/hbm.25314 (PMC7978137; doi:10.1002/hbm.25314)

Supplementary Methods

# HCP Demographics

| **Family Size** | **Count** |
| --- | --- |
| 1 | 74 |
| 2 | 162 |
| 3 | 178 |
| 4 | 35 |
| 5 | 3 |
| 6 | 1 |

| **Ethnicity** | **Count** |
| --- | --- |
| White | 734 |
| Black or African American | 176 |
| Hispanic/ Latino | 95 |
| Asian/ Nat. Hawaiian/ Other Pacific Is. | 61 |
| More than one | 22 |
| Unknown or Not Reported | 3 |
| Am. Indian/ Alaskan Nat. | 2 |

| **Age Group** | **Count** |
| --- | --- |
| 22-25 | 227 |
| 26-30 | 477 |
| 31-35 | 377 |
| 36+ | 12 |

# Bivariate SOLARIUS Outputs

PC1 (Negative Disposition) Bivariate SOLARIUS Analysis with iFC Phenotypes.

| PC1 (Negative Disposition) Correlation Variable | RhoP Est | RhoP P-val | RhoE Est | RhoE P-val | RhoG Est | RhoG P-val |
| --- | --- | --- | --- | --- | --- | --- |
| BST - Superficial Amygdala iFC | 0.012 | 0.700 | -0.026 | 0.725 | 0.197 | 0.559 |
| BST - Laterobasal Amygdala iFC | -0.002 | 0.943 | 0.027 | 0.702 | -0.303 | 0.651 |
| BST - CeA Amygdala iFC | 0.024 | 0.463 | -0.002 | 0.981 | NA | NA |
| BST - Centromedial Amygdala iFC | -0.008 | 0.80 | 0.067 | 0.356 | -0.349 | 0.270 |

PC2 (Alcohol Use) Bivariate SOLARIUS Analysis with iFC Phenotypes

| PC2 (Alcohol Use) Correlation Variable | RhoP Est | RhoP P-val | RhoE Est | RhoE P-val | RhoG Est | RhoG P-val |
| --- | --- | --- | --- | --- | --- | --- |
| BST - Superficial Amygdala iFC | -0.036 | 0.270 | -0.278 | 0.699 | -0.077 | 0.811 |
| BST - Laterobasal Amygdala iFC | -0.012 | 0.702 | -0.055 | 0.427 | 0.406 | 0.529 |
| BST - CeA Amygdala iFC | -0.013 | 0.703 | 0.091 | 0.135 | NA | NA |
| BST - Centromedial Amygdala iFC | 0.001 | 0.981 | 0.047 | 0.512 | -0.205 | 0.505 |

No phenotypic, environmental, or genetic correlations were observed between the principal components and the iFC variables. For the BST-CeA Amygdala iFC variable, RhoG could not be estimated because the heritability estimate of the variable is 0 (See Table 6).

# Mean Fractional Displacement Correlations


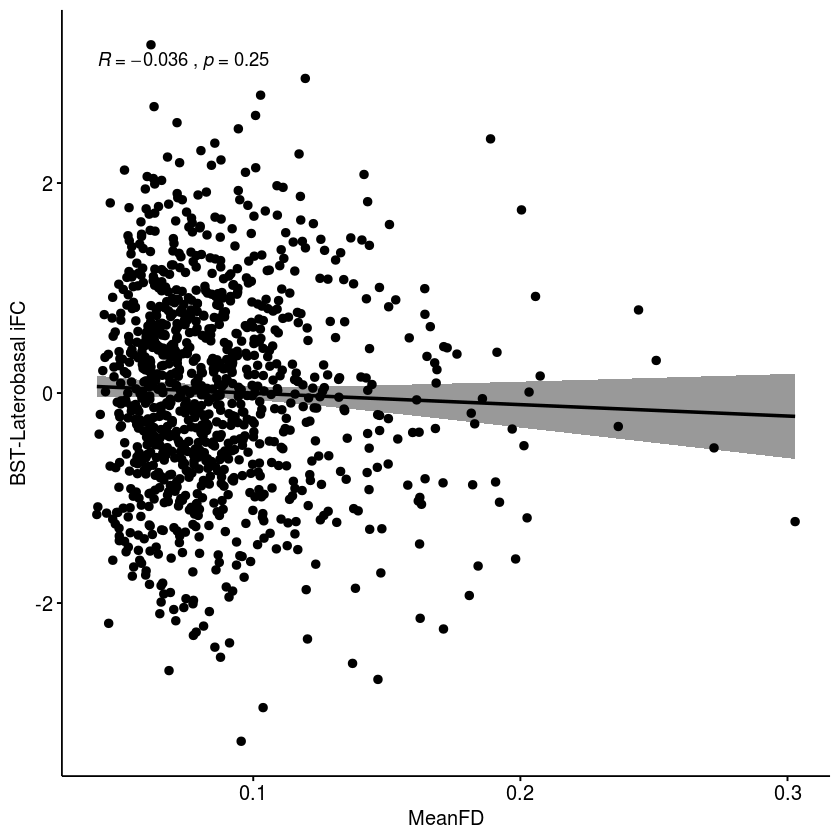

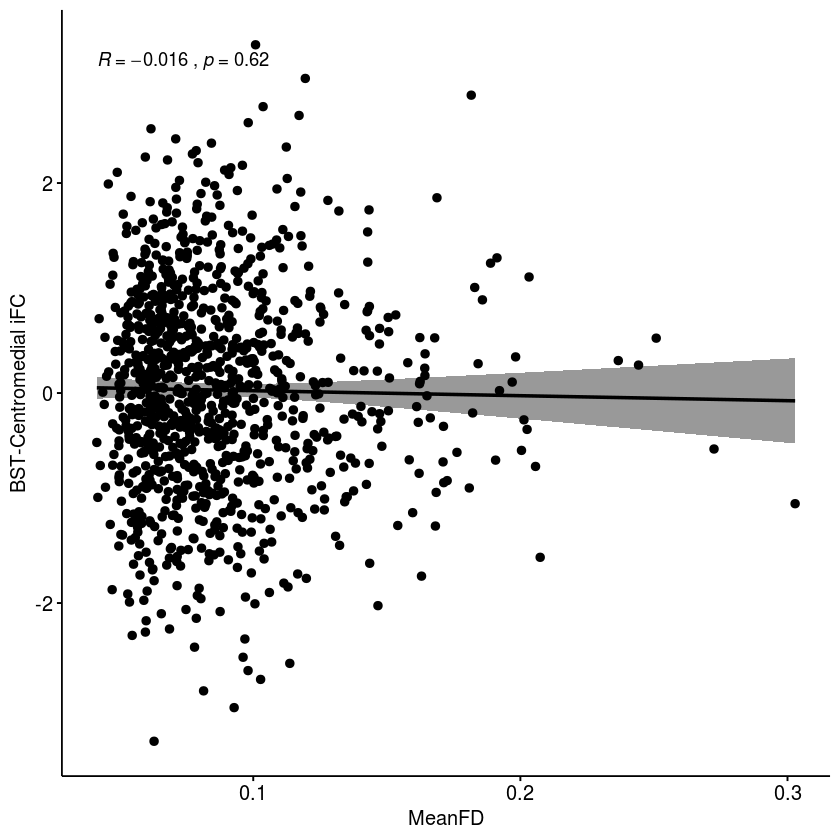

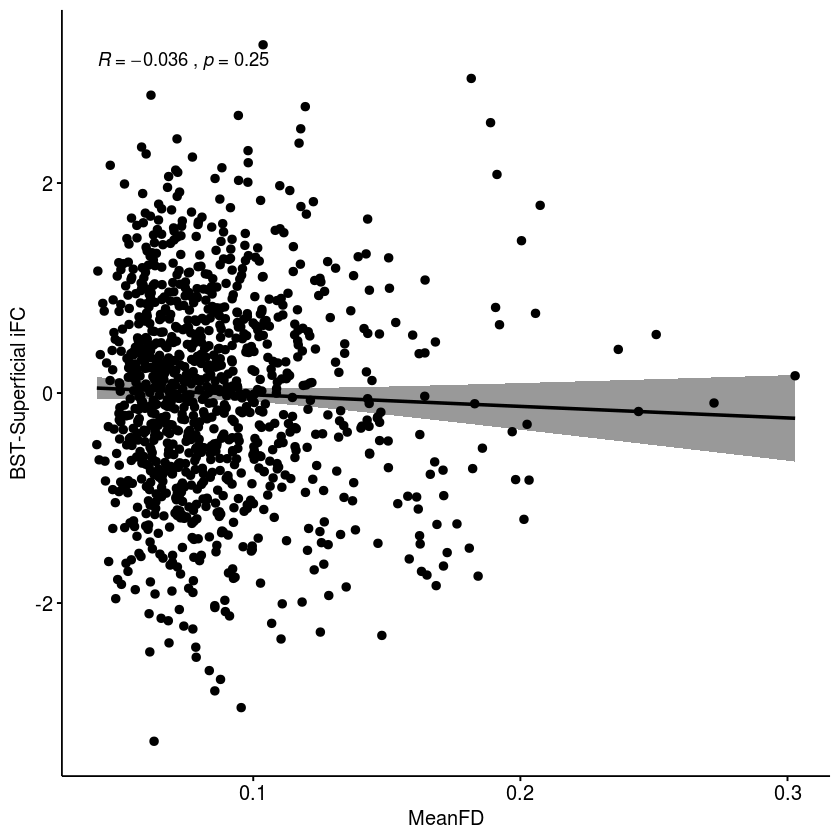

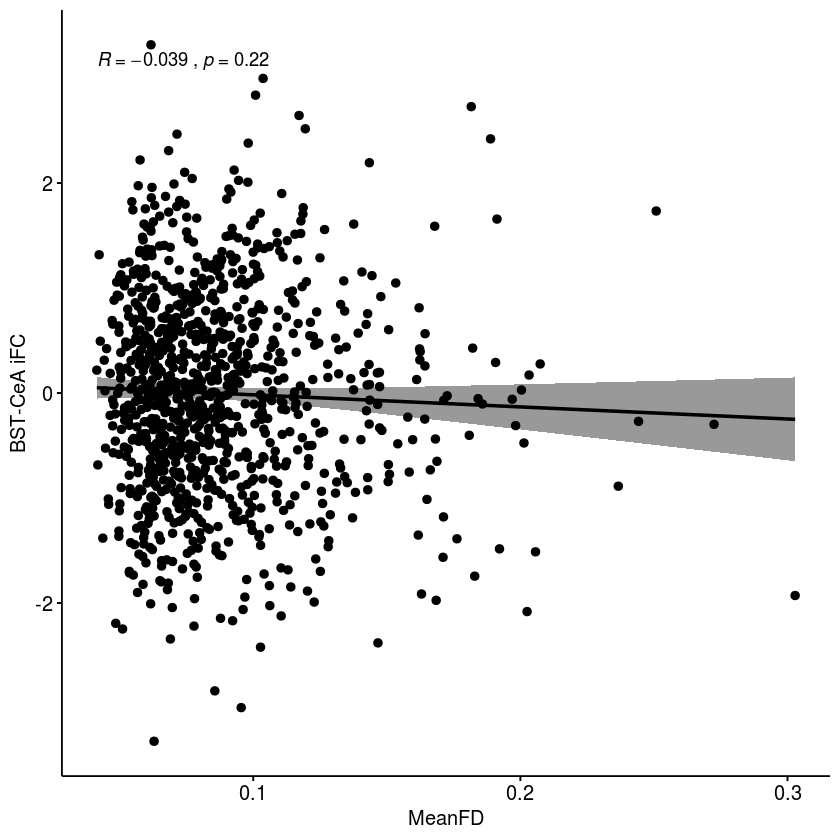

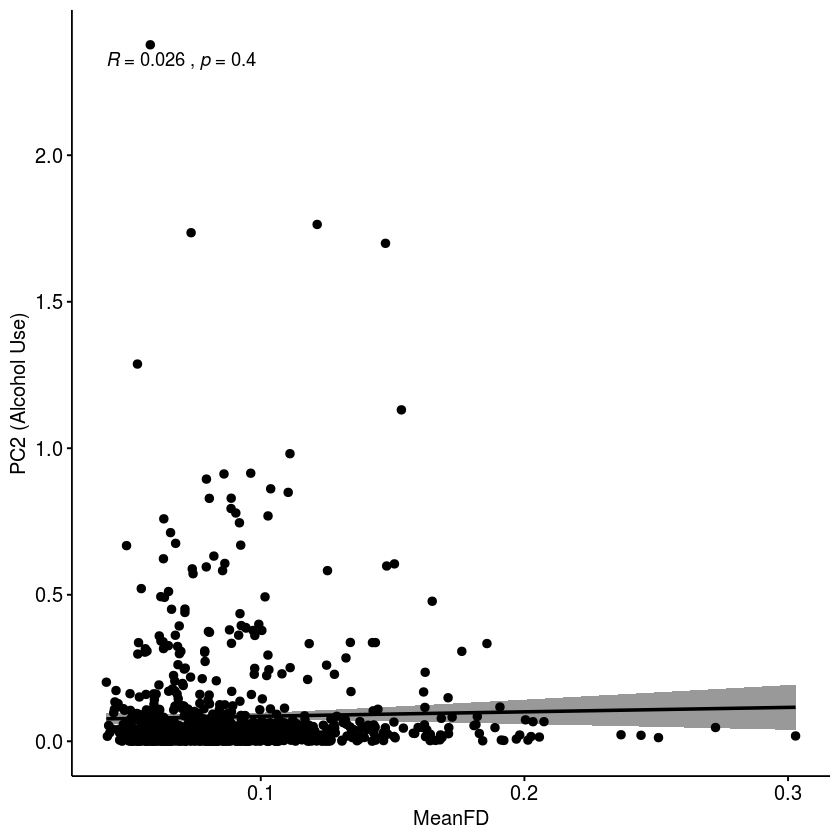

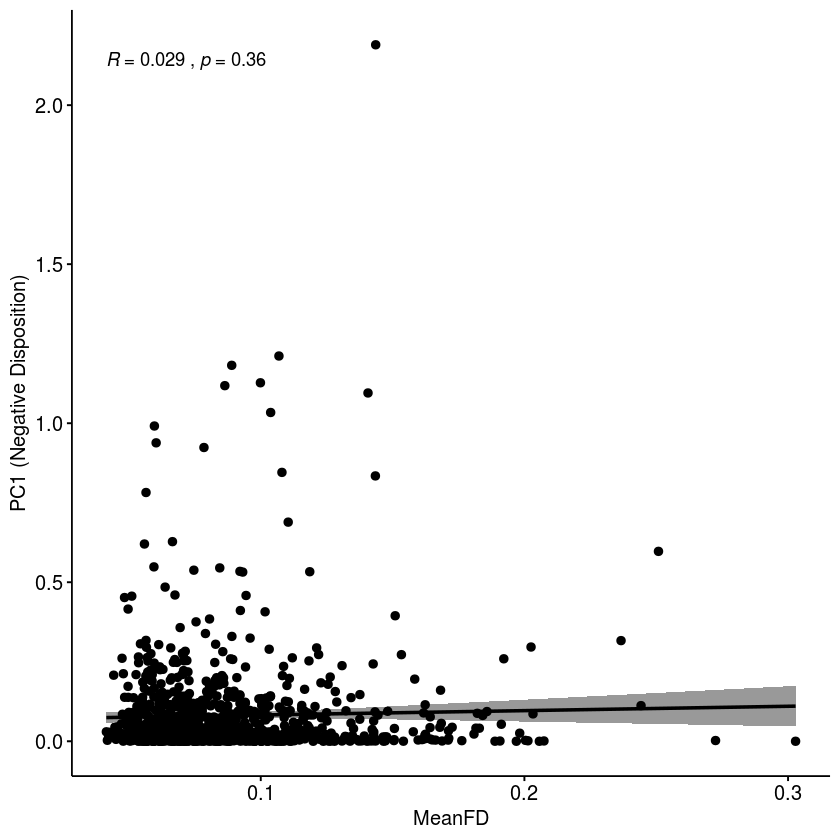

Supplement: Supplementary file 1 — Appendix S1: Supplementary Information [file HBM-42-1594-s001.docx]
